# Late phonological development in Williams syndrome

**DOI:** 10.3389/fpsyg.2022.992512

**Published:** 2022-11-16

**Authors:** Vanesa Pérez, Verónica Martínez, Eliseo Diez-Itza

**Affiliations:** ^1^LOGIN Research Group, University of Oviedo, Oviedo, Spain; ^2^Escuelas Universitarias Gimbernat, University of Cantabria, Torrelavega, Spain

**Keywords:** Williams syndrome, phonological development, intellectual disability, spontaneous speech assessment, phonological processes, atypical language development, neurodevelopmental genetic disorders

## Abstract

Williams syndrome is a neurodevelopmental genetic disorder characterized by a unique phenotype, including mild to moderate intellectual disability and an uneven neuropsychological profile of relative strengths and weaknesses. Language structure components (i.e., phonology, morphosyntax, and vocabulary) have been considered an area of specific ability compared to pragmatic language use. However, research on phonological development in Williams syndrome is very scarce, and it suggests atypical patterns. Therefore, the aim of the present study was to explore the profiles of late phonological development in Spanish-speaking children, adolescents, and adults with Williams syndrome, based on the analysis of five classes of processes (Syllable Structure, Substitution, Omission, Assimilation, and Addition) in spontaneous speech. The phonological profiles of seven children (aged 3–8 years), and seven adolescents and young adults (aged 14–25 years) with Williams syndrome were compared with two normative groups of typically developing (TD) children at different stages of late phonological development (aged 3 and 5 years). The frequency of phonological processes in the group of children with Williams syndrome was similar to that of 3-year-old TD children, which suggests that they would be in the first stage of late phonological development (expansion stage). The group of older individuals with Williams syndrome showed a much lower frequency of processes, similar to that of 5-year-old TD children in the last stage of phonological development (resolution stage). However, their phonological processes appeared to be persistent and independent of chronological age. Furthermore, asynchronies in quantitative and qualitative profiles (relative frequency) indicated atypical and complex trajectories in late phonological development, which cannot be described as simply delayed or protracted. Remarkable individual differences were observed, especially in the group of adolescents and adults with Williams syndrome, although the majority of cases conformed to the modal profiles of their groups. A major tendency for Omission, including final consonant deletion, may be considered atypical and specific to Williams syndrome at all ages. The results of the present study raise the need for continued and appropriate phonological assessment and treatment for people with Williams syndrome across the lifespan.

## Introduction

Williams syndrome (WS) is a neurodevelopmental disorder caused by a heterozygous deletion of between 26 and 28 genes on chromosome 7q11.23 (Pérez Jurado, 2003). The WS physical phenotype includes a distinctive facial appearance, hoarse voice, and sound sensitivities (hyperacusis, odynacusis, auditory allodynia, and auditory fascinations) (Kozel et al., 2021). Individuals with WS may show mild-to-moderate intellectual disability in conjunction with a distinct neurocognitive profile of relative strengths and weakness (Bellugi et al., 2000). Several studies have identified specific deficits in executive functioning (working memory, attentional abilities, and inhibition), problem-solving, and visuospatial skills (Camp et al., 2016; Heiz and Barisnikov, 2016; D’Souza et al., 2020). In contrast, auditory processing and face recognition are strengths in the WS profile (D’Souza et al., 2015; Miezah et al., 2020). Akin to the uneven cognitive profile, they also appear to show relative strengths and weaknesses in the motor profile, in the context of persisting fine and gross motor difficulties into childhood and adulthood (Mayall et al., 2021). Behavioral and emotional problems (attention, anxiety, and a range of social problems) have been also reported, together with a unique prosocial personality characterized by overfriendliness, a strong drive to approach strangers, gregariousness, bias toward positive affect, and heightened social engagement yet difficult peer interactions (Järvinen et al., 2013; Pérez-García et al., 2017). Special difficulties in adaptive behavior related to personal autonomy have also been described (Kirchner et al., 2016).

Language was first described as being selectively preserved and dissociated from other cognitive functions (Bellugi et al., 1988), although further research noted that language skills in individuals with WS were not intact and had complex interrelations with cognitive abilities (Mervis et al., 2004; Mervis and Becerra, 2007). Superior verbal skills reported in individuals with WS may be explained in terms of asynchronous trajectories of development with verbal ability progressing at a faster rate than non-verbal ability (Jarrold et al., 2001). In the same vein, language also shows asymmetrical development across different levels with varying outcomes in respect to what is expected for chronological and mental age (Brock, 2007). Pragmatic ability is an area of relative weakness, both in narrative and conversational settings (Stojanovik et al., 2001; Reilly et al., 2004; Stojanovik, 2006; Diez-Itza et al., 2018, 2022). In contrast, structural aspects of language have been described as relative strengths in the WS linguistic profile. Morphosyntactic abilities had been considered selectively spared (Clahsen et al., 2004), although this assumption was challenged in several studies indicating some degree of atypical morphological processing (Thomas et al., 2001; Boloh and Ibernon, 2010; Benítez-Burraco et al., 2017; Diez-Itza et al., 2017). Receptive vocabulary is also an area of relative strength in people with Down syndrome, but only for concrete vocabulary (Mervis and John, 2008; Garayzábal et al., 2014; Moraleda and López, 2020). Regarding lexical production, a tendency to use rare words and an atypical pattern of semantic categorization has been reported (Bellugi et al., 1994; Purser et al., 2010).

The phonological level is often considered another area of strength in the WS linguistic profile, although very few studies have directly assessed it. Most previous research focuses on phonological fluency, short-term memory (STM), phonological perception, and phonological awareness and processing (Vicari et al., 1996a,b; Volterra et al., 1996; Majerus et al., 2003; Majerus, 2004). Different studies have also been conducted on prosodic skills and their specific characteristics in the WS profile (Stojanovik, 2010; Martínez-Castilla et al., 2012). Only a few more recent studies have addressed phonological production in individuals with WS, although spontaneous speech was not analyzed but rather, words elicited from articulation tests (Hidalgo, 2019; Huffman, 2019). In general, both direct studies of production and those of phonological processing or prosody show that these skills are not fully preserved and that difficulties persist into adolescence and adulthood. However, in late phonological development, individuals with WS reach more advanced stages than other neuroevolutionary genetic syndromes, such as WS duplication syndrome, Smith Magenis syndrome, Down syndrome, and Fragile X syndrome (Mervis et al., 2015; Huelmo et al., 2017; Hidalgo and Garayzábal, 2019; Diez-Itza et al., 2021).

The existence of within-domain dissociations within the linguistic domain in WS, as well as specific developmental trajectories and atypical features, especially in the case of morphology, has been widely discussed (Karmiloff-Smith et al., 1997; Karmiloff-Smith, 1998; Diez-Itza et al., 2017). Phonological development provides a better example of emergent complexity, i.e., the changing nature of a complex system over time, revealing principles and milestones across languages (Davis and Bedore, 2013; McLeod and Crowe, 2018). The study of late stages in phonological development also suggests that the underlying dynamics are complex, from system expansion at around 3 years of age to its resolution at 5 years of age, which does not directly correspond to lexical production (Diez-Itza et al., 2001; Diez-Itza and Martínez, 2004). In this context, it could be discussed whether the alterations respond to a mere quantitative delay compared to typical development or whether they present trajectories specific to each disorder or syndrome (Rose and Inkelas, 2011). In this sense, the existence of protracted phonological development has been suggested in those cases with developmental trajectories that tend to converge late with those of typical development (Bernhardt and Stemberger, 2017; Vergara et al., 2021).

Both quantitative and qualitative differences could also depend on the age of the WS individuals studied. This question was addressed in one of the few studies that directly assessed the consonant articulation accuracy in two groups of English-speaking WS individuals (younger children: aged 4–9 years; older children and adolescents: aged 10–17 years) administered a Test of Articulation (Huffman, 2019). Consonant production accuracy was below expectations in both groups, but it was significantly higher for older children and adolescents. Patterns of articulatory accuracy in the group of younger children with WS were similar to the patterns of typically developing (TD) children, which means that articulation was significantly more accurate for early-developing consonants, followed by middle-developing consonants, and less accurate for late-developing consonants. In the group of older children and adolescents, all the early-developing consonants were correct, but this was not the case for middle- and late-developing consonants, where a similar proportion of articulatory accuracy was found. Manner-of-production was one of the sources of variation in articulatory accuracy, with Nasal and Stop consonants being significantly more accurate than Fricative and Approximant consonants in both groups. Although the patterns were similar, the older individuals showed quantitative growths: Nasal and Stop consonants reached full accuracy, and Fricative and Approximant consonants increased their accuracy by 50% to almost 90% of correct production. Articulatory accuracy of consonant clusters was also assessed and showed a sharp increase of almost 100% in the group of older children and adolescents with WS, and quite different patterns concerning particular vocal tract planes of movement in the control for articulatory accuracy.

The phonological production of Spanish-speaking individuals with WS between 4 and 31 years of age, compared with that of other syndromes, was also investigated by Hidalgo (2019) from the perspective of the phonological processes of simplification described by Bosch (2004) in TD children aged 3–7 years and the late stages of phonological development (expansion, stabilization, and resolution) established by Diez-Itza and Martínez (2004). From an articulation test, she observed that beyond the age of 6 years, phonetic and phonological repertoires were acquired by children with WS, although in some adolescents and adults, processes related to rhotic consonants persisted. The most frequent syllabic structure processes were cluster reduction (attacks and complex nuclei) and metathesis, and in a lower percentage, unstressed syllable omission, and addition, while reduplication and final consonant deletion processes were absent. In the case of segmental processes, the most frequent were absence or backing of rhotics, and in a lower percentage backing and deaffrication of other consonants, as well as assimilation processes.

Regarding phonological fluency, initial studies suggested that this is preserved in the WS linguistic profile, with children and adolescents with WS aged 4–15 years scoring better than their mental age-matched TD controls on a phonological fluency test without semantic involvement (Volterra et al., 1996). Based on these results, it was hypothesized that if only the phonological aspects of language develop at a normal rate while grammatical and lexical-semantic components remain impaired, it is because there is a dissociation between normal short-term and impaired long-term verbal memory in WS (Vicari et al., 1996b). Furthermore, performance in a word span task revealed comparable effects of phonological similarity and length to those observed in TD children, while the effect of frequency was significantly lower in WS participants, which was interpreted as the result of impaired access to lexical-semantic knowledge (Vicari et al., 1996a). Thus, a complex pattern of dissociation in linguistic processing and “atypical” development of WS children was revealed. It is important to note that the strength in phonology that these studies revealed is in any case relative since they compare individuals with WS with children of equal mental age but of much younger chronological age. Moreover, phonological development culminates in TD before the age of 9 years, whereas lexical development is open-ended.

The repetition of pseudowords has also contributed to the study of STM, showing that individuals with WS continue to rely strongly on phonological STM in the acquisition of new words, which is observed in 4-year-old but not in 5-year-old children (Grant et al., 1997). Phonological perception skills according to a nonsense syllables repetition test were comparable to those of TD participants with the same chronological age (range: 11–52 years) (Böhning et al., 2002). In a group of four children with WS who were administered both a word and pseudoword repetition test, their relative strength in STM was also confirmed to be comparable to that of children of the same chronological and verbal age in many respects, especially in the case of pseudowords where the support of phonological and lexico-semantic knowledge was minimized (Majerus et al., 2003).

In addition to word span and non-word repetition, phonological processing and phonological awareness skills were also studied in a group of children, adolescents, and adults with WS, which were compared with those of a group of TD children (mean age: 6.9), with differences emerging only in the phoneme deletion subtest (Laing et al., 2001). However, when the control groups were of the same chronological age or a verbal age closer to their chronological age, differences were observed in most measures of phonological awareness (Majerus et al., 2003). These results were explained by impairment at the level of the phonological representation (less finely grained) and the lexical-semantic representation (suggesting an abnormally structured network).

Phonological development is also often related in the early stages to motor aspects, as is the case with babbling. It has been claimed that the delay in the onset of canonical babbling and the first words observed in infants with WS is due to a delay in the acquisition of early motor milestones (Masataka, 2001). These findings are consistent with Velleman et al. (2006) who also observed delays in prelinguistic vocal development in six toddlers with WS. The postverbal onset of declarative gestures has also been linked with an atypical path of language development (Becerra and Mervis, 2019). An atypical accelerated trajectory of phonological development in two children with WS aged 5 was described by Martínez et al. (2014). At later stages, individuals with WS tend to present few phonological errors, which contrasts with the fact that difficulties in planning and coordinating oral-motor praxis in adolescents and adults with WS seem to persist (Krishnan et al., 2015).

Most studies, however, have not been conducted using developmental designs or naturalistic methodologies. Levy and Eilam (2013) analyzed extended spontaneous conversations in a mixed longitudinal study of two groups of children with WS and DS across five stages of morphophonological development. They concluded that there is a late-onset in both groups, determining atypical trajectories, which tend to show greater syndromic specificity at later stages of development. Capirci et al. (1996) and Diez-Itza et al. (1998), in longitudinal case studies of children with WS, found atypical phonological errors in conversational speech. The only recent study to our knowledge that addresses some aspects related to phonological production in spontaneous speech is that of Hargrove et al. (2012), who observed that adolescents with WS, although maintaining similar levels of intelligibility to their age peers, present a significantly lower rate of phonological accuracy, reaching more than 3% of incorrect words. They also found, like previous studies, a significantly slower speech rate in individuals with WS (Semel and Rosner, 2003; Setter et al., 2007; Crawford et al., 2008). However, their aims were not focused on the detailed analysis of phonology, nor did they offer a developmental perspective.

Several studies of late phonological development in TD Spanish-speaking children have been conducted based on cross-sectional designs. Aguilar and Serra (2003) and Bosch (2004) devised articulation tests and administered them to deliver normative data from children aged 3–7, including age of acquisition of the phonemic inventory and common processes at the different age stages. Diez-Itza et al. (2001), Diez-Itza and Martínez (2004), and Martínez (2010) registered and analyzed spontaneous speech corpora computing the frequency and the percentage distribution of phonological processes in children aged 3–5. An explicit aim of these analyses was to describe stages of phonological development as in previous studies by Ingram (1976) and Grunwell (1981). However, beyond a taxonomic description of processes at the different stages, the research by Diez-Itza and colleagues looked for quantitative and qualitative differences and non-linear trajectories of development. They found a reduction of the frequency of processes and changes in their relative distribution as age increased, suggesting three stages in late phonological development: expansion (age 3), stabilization (age 4), and resolution (age 5). Within the same theoretical and methodological framework, the present study aimed to further advance in a detailed description of late phonological development in children, adolescents and young adults with WS.

### Objectives

The main objective of the present study was to explore the profiles of late phonological development of Spanish-speaking individuals with WS to determine change across developmental stages and whether specific features would be exhibited. The profiles were based on the analysis of five classes of processes (Syllable Structure, Substitution, Omission, Assimilation, Addition) in spontaneous speech. The frequency and percentage distribution of processes were calculated, and modal profiles and outliers were determined by cluster analysis. It was hypothesized that late phonological development in WS follows the stages of typical development (i.e., expansion, stabilization, and resolution) and that phonological patterns show not only quantitative but also qualitative differences. To assess these hypotheses, the phonological profiles of children (aged 3–8), and adolescents and young adults (aged 14–25) with WS were compared with normative groups of TD preschool children at two stages of late phonological development (aged 3 and 5 years).

## Materials and methods

### Participants

The participants were 14 monolingual Spanish-speaking individuals with WS divided into two age groups (see Table 1): the first group (WS1) were children (chronological age: *M* = 5.8; *SD* = 1.6); the second group (WS2) were adolescents and young adults (chronological age: *M* = 19.6 years; *SD* = 3.7). They had been previously diagnosed by the molecular genetic test fluorescence *in situ* hybridization (FISH) and presented the characteristic phenotype. Parents and legal guardians provided informed consent for the participants to take part in the study.

**TABLE 1:** Gender, chronological and verbal age, and education of the participants with Williams syndrome.

Group	Case	Gender	CA	VA	Education
WS1	S1	Male	3.7	2.5	Regular school
	S2	Female	4.5	2.8	Regular school
	S3	Male	5.5	3.11	Regular school
	S4	Female	5.5	2.11	Regular school
	S5	Male	5.5	3.4	Regular school
	S6	Female	7.9	5.1	Regular school
	S7	Female	8.2	5.2	Regular school
WS2	S8	Male	14.4	10.1	Special school
	S9	Male	15.3	9.6	Regular school
	S10	Female	18.8	14.4	Vocational training
	S11	Female	19.11	8.6	Occupational center
	S12	Female	20.8	11.8	Occupational center
	S13	Female	23.3	8.8	Special school
	S14	Female	25.8	7.2	Occupational center

CA, chronological age; VA, verbal age.

To assess verbal lexical age and its relationship with phonological development, the participants were administered the Peabody Picture Vocabulary Test (Dunn et al., 2010): WS1 verbal age (*M* = 3.6; *SD* = 1.1) and WS2 verbal age (*M* = 10; *SD* = 2.4).

Normative data on the late phonological development of TD children were obtained from Martínez (2010), who established three stages in late phonological development (expansion, stabilization, and resolution) from 3.0 to 5.11. This study provides normative data in Spanish about phonological processes with the same methodology of spontaneous speech analysis as the present study. Thus, the WS1 and the WS2 groups were matched respectively with the group of younger children in the expansion stage (TD1) and the group of older children in the resolution stage (TD2) based on the frequency of processes. The TD1 normative group consisted of 40 children (20 girls and 20 boys; chronological age: *M* = 3.3 years; *SD* = 0.2); and the TD2 normative group also consisted of 40 children (20 girls and 20 boys) (chronological age: *M* = 5.8 years*; SD* = 0.3).

The participants with WS and TD children in the normative groups belonged to urban middle classes based on their district of residence within the Principality of Asturias and Cantabria (Spain), where a standard variant of Spanish (Castilian) is spoken.

### Instruments and procedure

The RETAMHE methodology, short for Recording, Transcription, and Analysis of Spontaneous Speech Samples (Diez-Itza, 1992; Diez-Itza et al., 1999), was used to obtain the spontaneous speech samples. Speech samples were collected via audio-visual recordings of dyadic conversations between each participant and a researcher, with an estimated duration of 45 min in natural settings, and which are part of larger corpora within the Syndroling Project (Diez-Itza et al., 2014). The researcher, who was familiar with the participants, introduced some degree of standardization by proposing common themes to all participants, according to the procedures developed by Abbeduto et al. (1995). The topics included telling a story, a visit to the doctor, a birthday party, talking about friends and family, weekend and daily activities, trips, and hobbies with variations among participants, following the spontaneous flow of conversation.

These conversations were transcribed in CHAT (Codes for the Human Analysis of Transcripts) format and analyzed with the FREQ program, one of the CLAN (Computerized Language Analysis) software programs, both provided by the CHILDES Project (MacWhinney, 2000). Each transcription was completed by a trained researcher and reviewed by two other researchers independently. Difficulties detected were analyzed jointly by the three investigators and discrepancies were resolved by the principal investigator. A total of 40,634 word tokens, 9,934 word types, and 2,806 phonological processes were analyzed, while 38 words were considered unintelligible.

The categories system proposed by Ingram (1976) and adapted by Diez-Itza et al. (2001) was used to code the phonological processes (PHO). The phonological processes were analyzed and classified into one of the following classes: Syllable Structure (SYS), Substitution (SBT), Omission (OMI), Assimilation (ASM), and Addition (ADD). In turn, each of these classes was divided into different subclasses of processes. Thus, SYS processes included Consonant Cluster Reduction (CCR), Final Consonant Deletion (FCD), Vowel Cluster (diphthong) Reduction (VCR), Unstressed Syllable Deletion (SYD), Metathesis (MTT), and Infrequent Processes (IFQ; Reduplication + Dissimilation + Analogy). SBT and OMI processes included Liquid (LIQ), Vowel (VOW), Fricative (FRC), Voiced Stop (VOS), Voiceless Stop (VLS), and Nasal (NSL). The following example illustrates the transcription and coding procedure according to the minCHAT format of the CHILDES Project.

*CHI: fesa [*] [: strawberry].

%err: **fesa = fresa $PHO:SYS:CCR**;

### Data analysis

Once the transcriptions were coded, the frequency of lexical variables was obtained using the FREQ program, that is, the total number of words produced (tokens) by each participant, as well as the count of different words (types) in each transcription. Next, the frequency of the classes and subclasses of phonological processes encoded was obtained with the same program.

Given the variability in the size of the spontaneous speech samples of each participant, the number of processes could not be directly used in the analyses. Therefore, to control differences introduced by the size of the samples, the frequency of processes was calculated through a Phonological Index (PI) (number of processes over 100 tokens).

In addition to the quantitative profile provided by the PI, qualitative distribution of the processes in each participant was analyzed. Therefore, the Relative Frequency (RF) was calculated, i.e., the percentage distribution of phonological processes by classes and subclasses. To calculate the RF, participants in each group who did not present phonological processes were not included in the analyses.

Between-group differences in PI and RF were analyzed using the non-parametric Mann–Whitney *U* test (expressed with the *Z* value) for independent samples, given that the distributions did not always approach normality according to the Shapiro–Wilk test. Additionally, the effect size was calculated by Cohen’s *d* using G*Power 3.1 statistical software. The *d* values are typically quantified as small (0.2), medium (0.5), and large (0.8) (Cohen, 1988). Spearman correlation was used to analyze the bivariate relationships between chronological age, verbal age, and PI.

In addition, individual similarities, and differences in the RF profiles of the classes and subclasses of phonological processes were explored by means of hierarchical cluster analysis, determining the modal cluster with the participants most similar to each other and best representing the group profile, additional clusters with participants that resemble each other, and extreme outlying cases.

Statistical analysis of the data was performed using SPSS software (Statistical Product and Service Solutions IBM SPSS Statistics 25.0).

## Results

### Phonological index

Table 2 reports the PI for each study group, including means for total processes and each class of processes. In the WS1 group, a strong positive correlation was found between chronological age and verbal age (*r*s = 0.94; *p* = 0.002), whereas the PI was negatively correlated with chronological age (*r*s = –0.78; *p* = 0.041); negative correlation between PI and verbal age failed to reach significance (*r*s = –0.64; *p* = 0.119). In the WS2 group, non-significant coefficients were obtained for negative correlation between chronological age and verbal age (*r*s = –0.54; *p* = 0.215); negative correlation between PI and verbal age (*r*s = –0.64; *p* = 0.119); and positive correlation between PI and chronological age (*r*s = 0.39; *p* = 0.383).

**TABLE 2:** Phonological processes index (total and by classes) means and standard deviations for groups, Mann–Whitney *U* test, and effect size.

	WS1	WS2	TD1	TD2	WS1 vs. WS2			WS1 vs. TD1			WS2 vs. TD2		
							
	PI-M (*SD*)	PI-M (*SD*)	PI-M (*SD*)	PI-M (*SD*)	*U* (*Z*)	*p*	*d*	*U* (*Z*)	*p*	*d*	*U* (*Z*)	*p*	*d*
TOT	18.4 (16.9)	1.8 (1.8)	13.3 (11.0)	1.4 (1.8)	2 (2.9)	0.01	1.4	125 (0.5)	0.65	0.4	110 (0.9)	0.37	0.2
SYS	10.3 (8.7)	1.1 (1.1)	7.7 (6.7)	0.8 (1.2)	2 (2.9)	0.01	1.5	120 (0.6)	0.55	0.3	99 (1.2)	0.22	0.3
SBT	3.9 (4.0)	0.4 (0.6)	3.9 (4.8)	0.3 (0.6)	4 (2.6)	0.01	1.2	138 (0.1)	0.95	0	105 (1.1)	0.29	0.2
OMI	2.7 (2.8)	0.2 (0.2)	0.9 (1.4)	0.1 (0.2)	5 (2.5)	0.01	1.3	74 (2.0)	0.05	0.8	70.5 (2.3)	0.02	0.5
ASM	0.7 (0.8)	0.1 (0.1)	0.5 (0.5)	0.1 (0.1)	10 (1.9)	0.06	1.1	125 (0.5)	0.65	0.3	114 (0.8)	0.42	0.1
ADD	0.4 (0.3)	0.04 (0.04)	0.3 (0.2)	0.1 (0.1)	0 (3.1)	0.01	1.7	100 (1.2)	0.23	0.4	119 (0.6)	0.52	0.8

PI-M, phonological index mean; TOT, total phonological processes index; SYS, syllable structure; SBT, substitution; OMI, omission; ASM, assimilation; ADD, addition; d, Cohen’s effect size.

Mann–Whitney *U* comparisons showed statistically significant differences between the WS groups in PI (total and in all classes of phonological process), with a large effect size except for ASM processes. The comparisons indicated that the WS1 group presented a higher frequency of all phonological processes except for ASM. No differences were observed between the WS1 and TD1 groups, or between the WS2 and TD2 groups, indicating that they were comparable in terms of the total frequency of processes and the frequency by class of processes, except for OMI. In the WS1 group, the PI for OMI processes was higher than in the TD1 group, and the Mann–Whitney *U* test yielded a statistically significant difference with a large effect size. In the WS2 group, the PI for OMI processes was higher than in the TD2 group, and the Mann–Whitney *U* test showed a statistically significant difference with a medium effect size.

Table 3 reports the PI for SYS subclasses of processes in each study group. Mann–Whitney *U* comparisons showed statistically significant differences between the WS groups, with a large effect size. Analyses showed significantly higher scores for the WS1 group for all SYS processes. No differences were observed between the WS1 and TD1 groups or between the WS2 and TD2 groups, indicating that they were comparable, except for MTT processes in the WS1 vs. TD1 group, and FCD processes in the WS2 vs. TD2 group. The PI for the MTT processes in the WS1 group was higher than in the TD1 group, and the PI for the FCD processes in the WS2 group was also higher than in the TD2 group. In both cases, the Mann–Whitney *U* test yielded statistically significant differences with a medium effect size. Additionally, statistically significant differences in IFQ were observed between the WS1 and TD1 groups with a medium effect size, and between the WS2 and TD2 groups with a small effect size.

**TABLE 3:** Syllable structure phonological processes index means and standard deviations for groups, Mann–Whitney *U* test, and effect size.

	WS1	WS2	TD1	TD2	WS1 vs. WS2			WS1 vs. TD1			WS2 vs. TD2		
							
	PI-M (SD)	PI-M (SD)	PI-M (SD)	PI-M (SD)	*U* (*Z*)	*p*	*d*	*U* (*Z*)	*p*	*d*	*U* (*Z*)	*p*	*d*
CCR	3.9 (3.2)	0.4 (0.5)	4.5 (4.6)	0.5 (1.0)	3 (2.8)	0.01	1.5	134 (0.2)	0.86	0.2	100 (1.2)	0.23	0.1
FCD	3.7 (3.2)	0.3 (0.4)	1.9 (2.3)	0.1 (0.2)	2 (2.9)	0.01	1.5	77 (1.9)	0.06	0.6	64 (2.4)	0.02	0.6
VCR	1.2 (1.2)	0.2 (0.1)	0.7 (0.7)	0.1 (0.2)	4 (2.6)	0.01	1.2	99 (1.2)	0.22	0.5	86 (1.6)	0.10	0.6
SYD	1.1 (1.3)	0.1 (0.1)	0.4 (0.6)	0.1 (0.1)	4 (2.6)	0.01	1.1	90 (1.5)	0.13	0.7	108 (1.0)	0.30	0.2
MTT	0.3 (0.3)	0.02 (0.02)	0.1 (0.3)	0.02 (0.04)	0 (3.1)	0.01	1.3	40 (3.1)	0.01	0.7	100 (1.6)	0.12	0
1IFQ	0.2 (0.2)	0.04 (0.1)	0.1 (0.1)	0.01 (0.03)	8.5 (2.1)	0.04	1.0	62 (2.5)	0.01	0.6	87.5 (2.1)	0.04	0.4

PI-M, phonological index mean; CCR, consonant cluster reduction; FCD, final consonant deletion; VCR, vowel cluster reduction; SYD, unstressed syllable deletion; MTT, metathesis; IFQ, infrequent processes; d, Cohen’s effect size.

Table 4 reports the PI for SBT subclasses of processes in each study group. Mann–Whitney *U* comparisons showed statistically significant differences between WS groups, with a large effect size, except for FRC. In the WS1 group, a higher frequency of SBT processes was observed in all subclasses except for FRC. No differences were observed between the WS1 and TD1 groups or between WS2 and TD2 groups, indicating that they were comparable, except for VOW and NSL substitutions. The PI for VOW substitution processes was much higher in the WS1 group than in the TD1 group, and in the WS2 group than in the TD2 group. In both cases, the Mann–Whitney *U* test yielded statistically significant differences with a large effect size. In addition, in the WS1 group, the PI for NSL substitution processes was higher than in the TD1 group, and the Mann–Whitney *U* test showed a statistically significant difference with a medium effect size.

**TABLE 4:** Substitution phonological processes index means and standard deviations for groups, Mann–Whitney U test, and effect size.

	WS1	WS2	TD1	TD2	WS1 vs. WS2			WS1 vs. TD1			WS2 vs. TD2		
							
	PI-M (SD)	PI-M (SD)	PI-M (SD)	PI-M (SD)	*U* (*Z*)	*p*	*d*	*U* (*Z*)	*p*	*d*	*U* (*Z*)	*p*	*d*
LIQ	1.2 (1.3)	0.1 (0.1)	1.2 (2.0)	0.2 (0.5)	0 (3.1)	0.01	1.2	117 (0.7)	0.49	0	110 (1.0)	0.31	0.3
VOW	0.9 (1.4)	0.1 (0.1)	0.1 (0.2)	0.02 (0.04)	5 (2.5)	0.01	0.8	66 (2.3)	0.03	0.8	74 (2.4)	0.02	1.1
FRC	0.7 (0.8)	0.3 (0.6)	1.8 (4.0)	0.1 (0.2)	10.5 (1.8)	0.07	0.6	128.5 (0.4)	0.73	0.4	113.5 (0.9)	0.36	0.4
VOS	0.6 (0.5)	0.03 (0.04)	0.4 (0.6)	0.02 (0.04)	2 (2.9)	0.01	1.6	102.5 (1.1)	0.26	0.4	120 (0.8)	0.45	0.3
VLS	0.4 (0.5)	0.01 (0.02)	0.3 (0.5)	0.01 (0.03)	3 (2.8)	0.01	1.1	105 (1.1)	0.29	0.2	121.5 (0.9)	0.37	0
NSL	0.2 (0.3)	0.01 (0.03)	0.1 (0.2)	0.02 (0.1)	4 (2.7)	0.01	0.9	71 (2.2)	0.03	0.4	134 (0.3)	0.78	0.1

LIQ, liquid; VOW, vowel; FRC, fricative; VOS, voiced stop; VLS, voiceless stop; NSL, nasal; d, Cohen’s effect size.

Table 5 reports the PI for the OMI subclasses of processes in each study group. Mann–Whitney *U* comparisons showed statistically significant differences between WS groups, with a large effect size, except for VOS omission processes. In the WS1 group, a higher frequency of OMI was observed in all subclasses, except for VOS consonants. Differences between the WS1 and TD1 groups were observed in all OMI subclasses, except for LIQ and VOS omissions, where both groups were comparable. The PI of the VOW, NSL, and FRC omission processes was much higher in the WS1 group than in the TD1 group. In all three subclasses, the Mann–Whitney *U* test yielded statistically significant differences with a large effect size. For VLS omission processes, the difference was also statistically significant, with a medium effect size. No differences were observed between the WS2 and TD2 groups, indicating that they were comparable, except for the LIQ and VOW omissions, where the PI in the WS2 group was higher. In both cases, the Mann–Whitney *U* test yielded statistically significant differences with a medium effect size.

**TABLE 5:** Omission phonological processes index means and standard deviations for groups, Mann–Whitney *U* test, and effect size.

	WS1	WS2	TD1	TD2	WS1 vs. WS2			WS1 vs. TD1			WS2 vs. TD2		
							
	PI-M (*SD*)	PI-M (*SD*)	PI-M (*SD*)	PI-M (*SD*)	*U* (*Z*)	*p*	*d*	*U* (*Z*)	*p*	*d*	*U* (*Z*)	*p*	*d*
LIQ	1.3 (1.5)	0.1 (0.1)	0.6 (1.0)	0.03 (0.1)	8 (2.1)	0.04	1.1	79 (1.9)	0.06	0.5	45 (3.7)	0.001	0.7
VOS	0.4 (0.5)	0.1 (0.1)	0.2 (0.4)	0.04 (0.1)	11 (1.8)	0.07	0.8	99 (1.3)	0.21	0.4	111 (1.2)	0.23	0.6
VLS	0.4 (0.6)	0.004 (0.01)	0.1 (0.1)	0.002 (0.01)	4 (2.8)	0.01	0.9	53.5 (3.1)	0.01	0.7	124 (1.4)	0.17	0.2
VOW	0.3 (0.3)	0.02 (0.03)	0.03 (0.1)	0 (0)	6 (2.4)	0.02	1.3	38.5 (3.7)	0.001	1.2	80 (4.2)	0.001	0.7
NSL	0.3 (0.3)	0.01 (0.02)	0.03 (0.1)	0.004 (0.02)	33 (2.7)	0.01	1.4	48.5 (3.3)	0.001	1.2	127 (0.9)	0.36	0.3
FRC	0.1 (0.1)	0 (0)	0.02 (0.04)	0.008 (0.03)	35 (2.6)	0.01	1	64 (3.1)	0.01	1.1	126 (0.9)	0.39	0.3

LIQ, liquid; VOS, voiced stop; VLS, voiceless stop; VOW, vowel; NSL, nasal; FRC, fricative; d, Cohen’s effect size.

### Relative frequency

**FIGURE 1:**
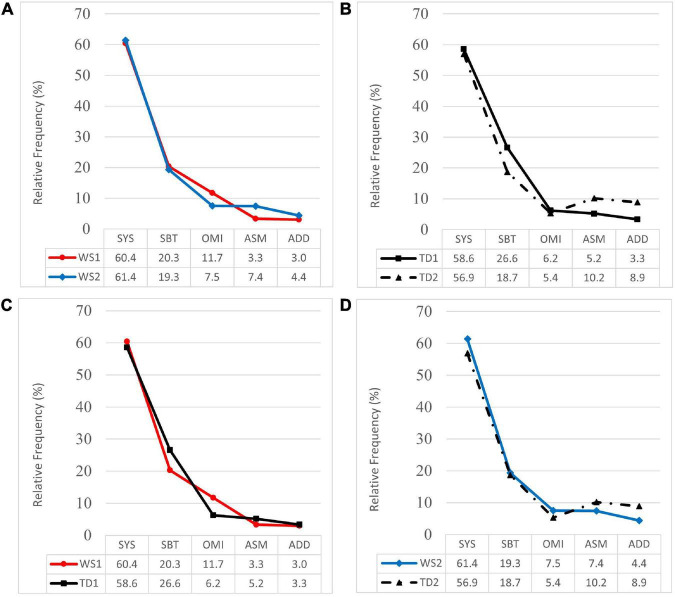
Profiles of relative frequency of processes by classes (SYS, syllable structure; SBT, substitution; OMI, omission; ASM, assimilation; ADD, addition) for WS groups and TD groups. **(A)** Profiles of WS1 and WS2 groups. **(B)** Profiles of TD1 and TD2 groups. **(C)** Profiles of WS1 and TD1groups. **(D)** Profiles of WS2 and TD2 groups.

In Figure 1, the compared profiles of RF, i.e., the percentage distribution, for processes by classes are shown. Figure 1A represents the profiles of WS1 and WS2 groups, which were very similar in terms of the percentage of the most frequent classes of processes (SYS, SBT). In the classes of OMI and ASM processes, the profiles of both groups intersected since the WS2 group showed a relatively lower percentage of OMI and a correspondingly higher percentage of ASM. However, the Mann–Whitney *U* test did not yield statistically significant differences: SYS (*U* = 22; *Z* = 0.32; *p* = 0.75; *d* = 0.1); SBT (*U* = 17; *Z* = 0.96; *p* = 0.34; *d* = 0.1); OMI (*U* = 12; *Z* = 1.60; *p* = 0.11; *d* = 0.8); ASM (*U* = 11; *Z* = 1.73; *p* = 0.09; *d* = 1.1); ADD (*U* = 24; *Z* = 0.06; *p* = 0.95; *d* = 0.3).

Figure 1B represents the compared profiles of normative groups TD1 and TD2 (*n* = 39), which were similar in terms of the percentage of SYS processes. The profile of the TD2 group showed a relatively lower percentage of SBT and OMI processes. In both classes, the Mann–Whitney *U* test showed statistically significant differences: SBT (*U* = 520; *Z* = 2.56; *p* = 0.01; *d* = 0.4); OMI (*U* = 577.5; Z = 2.07; *p* = 0.04; *d* = 0.1). Inversely, the profile of the TD2 group showed a relatively higher percentage of ASM and ADD processes, although no further statistically significant differences were observed: SYS (*U* = 773; *Z* = 0.07; *p* = 0.95; *d* = 0.1); ASM (*U* = 725; *Z* = 0.54; *p* = 0.59; *d* = 0.1); ADD (*U* = 695; *Z* = 0.84; *p* = 0.40; *d* = 0.6).

Figure 1C represents the compared profiles of WS1 and TD1 groups, where the profile of the WS1 group showed a higher percentage of OMI processes, and the Mann–Whitney *U* test yielded statistically significant differences: OMI (*U* = 69; *Z* = 2.13; *p* = 0.03; *d* = 0.9). The most frequent processes in both groups were SYS with similar percentages, while the profile of the WS1 group showed a relatively lower percentage of SBT and ASM processes, although no statistically significant differences were observed: SYS (*U* = 135; *Z* = 0. 15; *p* = 0.88; *d* = 0.1); SBT (*U* = 105; *Z* = 1.05; *p* = 0.30; *d* = 0.5); ASM (*U* = 134; *Z* = 0.18; *p* = 0.86; *d* = 0.4); ADD (*U* = 105; *Z* = 1.05; *p* = 0.30; *d* = 0.1).

Figure 1D represents the compared profiles of WS2 and TD2 groups (*n* = 39), which were similar in terms of the percentage of SYS and SBT processes. The profile of the WS2 group showed a relatively lower percentage of ASM and ADD processes, and a relatively higher percentage of OMI processes. However, the Mann–Whitney *U* test did not yield statistically significant differences: SYS (*U* = 124; *Z* = 0.37; *p* = 0.71; *d* = 0. 2); SBT (*U* = 104.5; *Z* = 0.99; *p* = 0.32; *d* = 0.03); OMI (*U* = 84; *Z* = 1.75; *p* = 0.08; *d* = 0.3); ASM (*U* = 99; *Z* = 1.19; *p* = 0.23; *d* = 0.2); ADD (*U* = 130; *Z* = 0.19; *p* = 0.85; *d* = 0.5).

**FIGURE 2:**
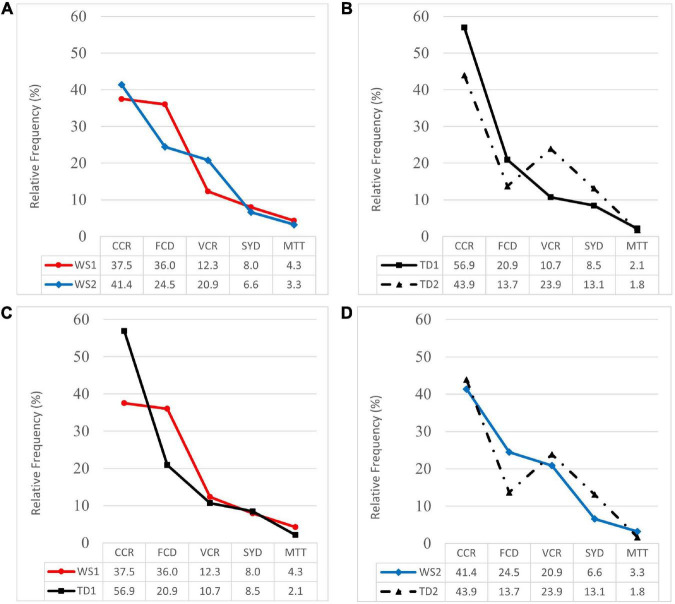
Profiles of relative frequency of syllable structure processes (CCR, consonant cluster reduction; FCD, final consonant deletion; VCR, vowel cluster reduction; SYD, unstressed syllable deletion; MTT, metathesis) for WS groups and TD groups. **(A)** Profiles of WS1 and WS2 groups. **(B)** Profiles of TD1 and TD2 groups. **(C)** Profiles of WS1 and TD1 groups. **(D)** Profiles of WS2 and TD2 groups.

In Figure 2, the compared profiles of RF, i.e., the percentage distribution, for the SYS subclasses of processes are shown. Figure 2A represents the profiles of WS1 and WS2 groups, which were very similar in terms of the percentage of the most frequent processes (CCR). In the subclasses of FCD and VCR processes, the profiles of both groups intersected since the WS2 group showed a relatively lower percentage of FCD and a correspondingly higher percentage of VCR, although the Mann–Whitney *U* test did not yield statistically significant differences: CCR (*U* = 19; *Z* = 0.70; *p* = 0.48; *d* = 0.3); FCD (*U* = 11; *Z* = 1.73; *p* = 0.09; *d* = 1.1); VCR (*U* = 12; *Z* = 1.6; *p* = 0.11; *d* = 0.9); SYD (*U* = 20; *Z* = 0.58; *p* = 0.57; *d* = 0.3); MTT (*U* = 17; *Z* = 0.96; *p* = 0.34; *d* = 0.3).

Figure 2B represents the compared profiles of normative groups TD1 and TD2 (*n* = 36). The profile of the TD2 group showed a relatively lower percentage in the most frequent subclasses of SYS processes (CCR, FCD) and in the less frequent subclass (MTT). In the three subclasses, the Mann Whitney *U* test showed statistically significant differences: CCR (*U* = 533.5; *Z* = 1.94; *p* = 0.05; *d* = 0.5); FCD (*U* = 478; *Z* = 2.55; *p* = 0.01; *d* = 0.4); MTT (*U* = 501; *Z* = 2.54; *p* = 0.01; *d* = 0.1). In addition, the profile of the TD2 group showed a relatively higher percentage of VCR and SYD processes, although no further statistically significant differences were observed: VCR (*U* = 586.5; *Z* = 1.40; *p* = 0.16; *d* = 0.6); SYD (*U* = 619; *Z* = 1.08; *p* = 0.28; *d* = 0.3).

Figure 2C represents the compared profiles of the WS1 and TD1 groups, where the WS1 group profile showed a relatively lower percentage of CCR processes and a relatively higher percentage of FCD and MTT processes. In the three subclasses, the Mann–Whitney *U* test yielded statistically significant differences: CCR (*U* = 49; *Z* = 2.72; *p* = 0.01; *d* = 1.3); FCD (*U* = 60; *Z* = 2.39; *p* = 0.02; *d* = 1.2); MTT (*U* = 56; *Z* = 2.56; *p* = 0.01; *d* = 0.6). Both profiles were similar in terms of the percentage of VCR and SYD processes: VCR (*U* = 108; *Z* = 0.96; *p* = 0.34; *d* = 0.2); SYD (*U* = 108; *Z* = 0.96; *p* = 0.34; *d* = 0.1).

Figure 2D represents the compared profiles of WS2 and TD2 groups (*n* = 36), where the profile of the WS2 group showed a relatively higher percentage of FCD processes and the Mann–Whitney *U* test yielded statistically significant difference: FCD (*U* = 67.5; *Z* = 1.99; *p* = 0.05; *d* = 0.7). The most frequent subclasses of SYS processes in both groups were CCR and showed similar percentages, while the profile of the WS2 group presented relatively lower percentages of VCR, SYD, and a relatively higher percentage of MTT processes, although no further statistically significant differences were observed: CCR (*U* = 120.5; *Z* = 0.18; *p* = 0.86; *d* = 0.1); VCR (*U* = 110; *Z* = 0.53; *p* = 0.60; *d* = 0.1); SYD (*U* = 119.5; *Z* = 0.23; *p* = 0.82; *d* = 0.4); MTT (*U* = 83; *Z* = 1.79; *p* = 0.07; *d* = 0.3).

**FIGURE 3:**
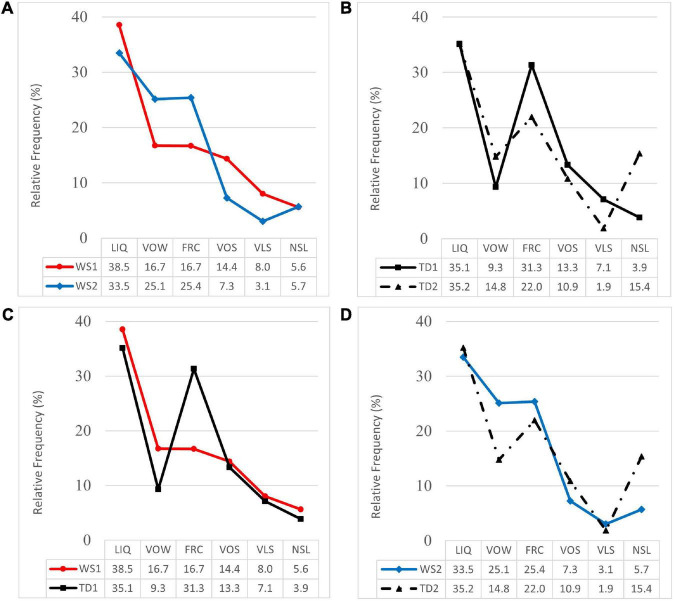
Profiles of relative frequency of substitution processes (LIQ, liquid; VOW, vowel; FRC, fricative; VOS, voiced stop; VLS, voiceless stop; NSL, nasal) for WS groups and TD groups. **(A)** Profiles of WS1 and WS2 groups. **(B)** Profiles of TD1 and TD2 groups. **(C)** Profiles of WS1 and TD1 groups. **(D)** Profiles of WS2 and TD2 groups.

In Figure 3, the compared profiles of RF for SBT subclasses of processes are shown. Figure 3A represents the profiles of the WS1 and WS2 groups. In the WS2 group profile, a relatively lower percentage of VLS substitutions and a relatively higher percentage of NSL substitutions were observed, and the Mann–Whitney *U* test yielded statistically significant differences: VLS (*U* = 6; *Z* = 2.42; *p* = 0.02; *d* = 0.7); NSL (*U* = 7; *Z* = 2.33; *p* = 0.02; *d* = 0.2). Further intersections in the profiles of both groups were observed, since the WS2 group showed relatively lower percentages of LIQ and VOS substitutions, and correspondingly higher percentages of VOW and FRC substitutions. However, these differences were not statistically significant: LIQ (*U* = 22; *Z* = 0. 32; *p* = 0.75; *d* = 0.2); VOS (*U* = 12; *Z* = 1.62; *p* = 0.11; *d* = 0.8); VOW (*U* = 24; *Z* = 0.06; *p* = 0.95; *d* = 0.4); FRC (*U* = 24; *Z* = 0; *p* = 1.0; *d* = 0.3).

Figure 3B represents the compared profiles of normative groups TD1 (*n* = 37) and TD2 (*n* = 25), where the TD2 profile showed relatively lower percentages of FRC, VOS, and VLS substitutions. In the three subclasses, the Mann–Whitney *U* test showed statistically significant differences: FRC (*U* = 329.5; *Z* = 1.93; *p* = 0.05; *d* = 0.3); VOS (*U* = 324.5; *Z* = 2.03; *p* = 0.04; *d* = 0.1); VLS (*U* = 241.5; *Z* = 3.42; *p* = 0.001; *d* = 0.8). The most frequent processes in both groups were LIQ substitutions showing similar percentages, while in the profile of the TD2 group relatively higher percentages of VOW and NSL substitutions were observed. However, these differences were not statistically significant: LIQ (*U* = 383. 5; *Z* = 1.14; *p* = 0.25; *d* = 0.01); VOW (*U* = 389; *Z* = 1.11; *p* = 0.27; *d* = 0.2); NSL (*U* = 433; *Z* = 0.49; *p* = 0.63; *d* = 0.5).

Figure 3C represents the compared profiles of the WS1 and TD1 groups (*n* = 37), where the WS1 profile presented relatively higher percentages of VOW and NSL substitution processes. In both subclasses, the Mann–Whitney *U* test showed statistically significant differences: VOW (*U* = 51; *Z* = 2.55; *p* = 0.01; *d* = 0.5); NSL (*U* = 72; *Z* = 1.95; *p* = 0.05; *d* = 0.4). The most frequent processes in both groups were LIQ substitutions showing similar percentages, while in the profile of the WS1 group a relatively lower percentage of FRC substitutions was observed. However, no further statistically significant differences were observed: LIQ (*U* = 120; *Z* = 0. 31; *p* = 0.76; *d* = 0.1); FRC (*U* = 87; *Z* = 1.37; *p* = 0.17; *d* = 0.7); VOS (*U* = 92; *Z* = 1.19; *p* = 0.23; *d* = 0.1); VLS (*U* = 107; *Z* = 0.73; *p* = 0.47; *d* = 0.1).

Figure 3D represents the compared profiles of the WS2 and TD2 groups (*n* = 25), which were similar in terms of the percentage of the most frequent processes (LIQ). The profile of the WS2 group showed a relatively higher percentage of VOW substitutions and a relatively lower percentage of NSL substitutions. However, the Mann–Whitney *U* test did not yield statistically significant differences: LIQ (*U* = 79; *Z* = 0.41; *p* = 0.69; *d* = 0.1); VOW (*U* = 60; *Z* = 1.38; *p* = 0.17; *d* = 0.4); FRC (*U* = 80; *Z* = 0.36; *p* = 0.72; *d* = 0. 2); VOS (*U* = 85.5; *Z* = 0.10; *p* = 0.92; *d* = 0.2); VLS (*U* = 80; *Z* = 0.47; *p* = 0.64; *d* = 0.2); NSL (*U* = 75; *Z* = 0.75; *p* = 0.45; *d* = 0.4).

**FIGURE 4:**
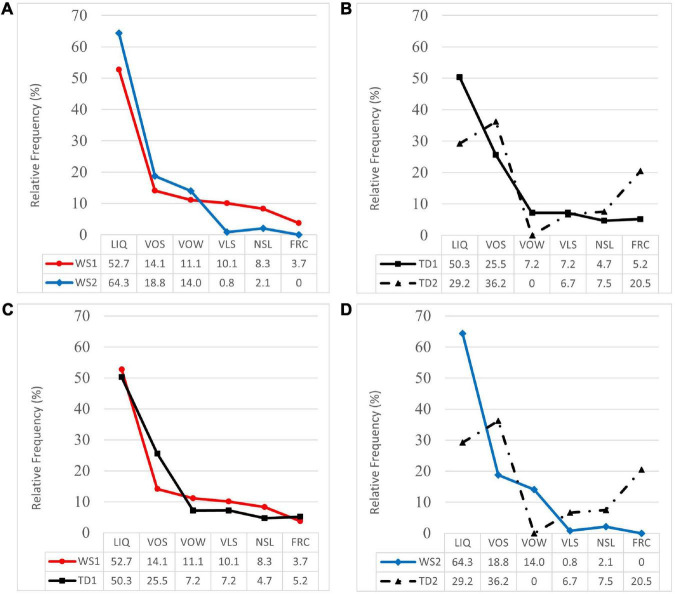
Profiles of relative frequency of omission processes (LIQ, liquid; VOW, vowel; FRC, fricative; VOS, voiced stop; VLS, voiceless stop; NSL, nasal) for WS groups and TD groups. **(A)** Profiles of WS1 and WS2 groups. **(B)** Profiles of TD1 and TD2 groups. **(C)** Profiles of WS1 and TD1 groups. **(D)** Profiles of WS2 and TD2 groups.

In Figure 4, the compared profiles of RF for the OMI subclasses of processes are shown. Figure 4A represents the profiles of WS1 and WS2 groups (*n* = 6), where the WS2 profile presented a relatively lower percentage of the less frequent OMI processes (VLS, NSL, FRC). In these subclasses, the Mann–Whitney *U* test yielded statistically significant differences: VLS (*U* = 4.5; *Z* = 2.48; *p* = 0.01; *d* = 1.6); NSL (*U* = 8.5; *Z* = 1.88; *p* = 0.06; *d* = 0.9); FRC (*U* = 6; *Z* = 2.44; *p* = 0.02; *d* = 0.7). In contrast, the WS2 profile showed relatively higher percentages of the most frequent OMI processes (LIQ, VOS, VOW). However, in these subclasses, no statistically significant differences were found: LIQ (*U* = 18; *Z* = 0.43; *p* = 0.67; *d* = 0.4); VOS (*U* = 20; *Z* = 0.15; *p* = 0.88; *d* = 0.2); VOW (*U* = 14.5; *Z* = 0.94; *p* = 0.35; *d* = 0.1).

Figure 4B represents the compared profiles of normative groups TD1 (*n* = 30) and TD2 (*n* = 15). In the TD2 profile, a relatively lower percentage of VOW omissions was observed, where the Mann–Whitney *U* test yielded statistically significant differences: VOW (*U* = 157.5; *Z* = 2.33; *p* = 0.02; *d* = 0.4). Further intersections in the profiles of both groups were observed, since the TD2 group showed relatively lower percentages of LIQ omissions, and correspondingly higher percentages of VOS and FRC omissions. However, these differences were not statistically significant: LIQ (*U* = 155.5; *Z* = 1.71; *p* = 0.09; *d* = 0.5); VOS (*U* = 215; *Z* = 0.25; *p* = 0.81; *d* = 0.3); VLS (*U* = 176.5; *Z* = 1. 61; *p* = 0.11; *d* = 0.02); NSL (*U* = 192.5; *Z* = 1.04; *p* = 0.30; *d* = 0.1); FRC (*U* = 202.5; *Z* = 0.75; *p* = 0.46; *d* = 0.5).

Figure 4C represents the compared profiles of WS1 and TD1 groups (*n* = 30), where the WS1 profile showed relatively higher percentages of VOW, VLS, and NSL omission processes, and a relatively lower percentage of FRC omissions. In these subclasses, the Mann–Whitney *U* test yielded statistically significant differences: VOW (*U* = 49; *Z* = 2.44; *p* = 0.02; *d* = 0.3); VLS (*U* = 56.5; *Z* = 2.12; *p* = 0.03; *d* = 0.2); NSL (*U* = 49.5; *Z* = 2.42; *p* = 0.02; *d* = 0.4); FRC (*U* = 56; *Z* = 2.35; *p* = 0.02; *d* = 0.1). The most frequent processes in both groups were LIQ omissions, with similar percentages, while the profile of the WS1 group presented a relatively lower percentage of VOS omissions, although no statistically significant differences were observed: LIQ (*U* = 104; *Z* = 0.02; *p* = 0.98; *d* = 0.1); VOS (*U* = 86.5; *Z* = 0.72; *p* = 0.47; *d* = 0.6).

Figure 4D represents the compared profiles of the WS2 (*n* = 6) and TD2 (*n* = 15) groups, where the profile of the WS2 group showed relatively higher percentages of LIQ and VOW omission processes. In both subclasses, the Mann–Whitney *U* test yielded statistically significant differences: LIQ (*U* = 20.5; *Z* = 2.00; *p* = 0.05; *d* = 1.0); VOW (*U* = 22.5; *Z* = 2.88; *p* = 0.01; *d* = 0.5). The WS2 profile showed relatively lower percentages of VOS, VLS, NSL, and FRC omissions, although no further statistically significant differences were observed: VOS (*U* = 38. 5; *Z* = 0.60; *p* = 0.55; *d* = 0.5); VLS (*U* = 41; *Z* = 0.61; *p* = 0.54; *d* = 0.3); NSL (*U* = 44; *Z* = 0.13; *p* = 0.90; *d* = 0.3); FRC (*U* = 33; *Z* = 1.37; *p* = 0.17; *d* = 0.5).

### Cluster analysis

**FIGURE 5:**
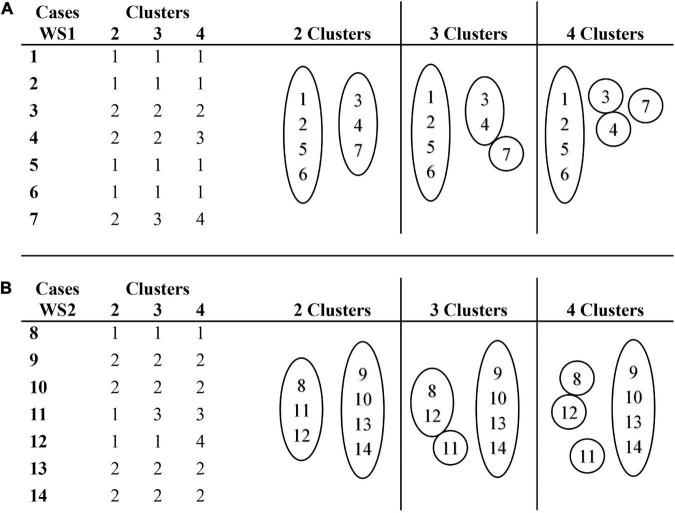
Cluster membership for a range of solutions (2, 3, and 4 clusters) for classes of processes. **(A)** Cluster membership of cases in WS1 group. **(B)** Cluster membership of cases in WS2 group.

In Figure 5, the clusters membership (solutions for 2, 3, and 4 clusters) indicate the individual similarities and differences in the RF profiles of classes of processes within the WS1 and WS2 groups. Figure 5A shows that, in the WS1 group, the profiles of cases 1, 2, 5, and 6 represent the modal profile, i.e., the predominant patterns. Cases 3, 4, and 7 diverge from that profile in two directions: they present a higher percentage of SYS processes diverging from the TD1 group; they also present a lower percentage of OMI processes converging with the TD1 group.

Figure 5B represents the clusters membership in the WS2 group, where it is observed that the profiles of cases 9, 10, 13, and 14 represent the modal profile. Cases 8, 11, and 12 diverge from that profile, diverging from the TD2 group, by presenting a higher percentage of SYS processes and a lower percentage of SBT processes.

**FIGURE 6:**
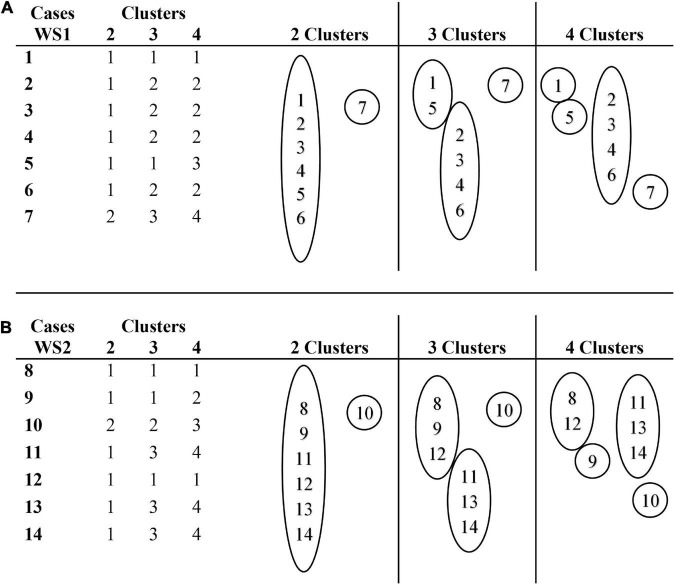
Cluster membership for a range of solutions (2, 3, and 4 clusters) for syllable structures processes. **(A)** Cluster membership of cases in WS1 group. **(B)** Cluster membership of cases in WS2 group.

In Figure 6, the clusters membership of the RF profiles of SYS subclasses of processes are shown. Figure 6A shows that the profiles of cases 2, 3, 4, and 6 represent the modal profile of the WS1 group, while case 7 is an extreme case because of its high percentage of FCD. Cases 1 and 5 diverge from the TD1 group by their lower percentage of CCR and a higher percentage of VCR processes.

Figure 6B represents the membership clusters in the WS2 group, where it is observed that the profiles of cases 11, 13, and 14 represent the modal profile, while case 10 is an extreme case, due to its low percentage of CCR, and its high percentage of FCD and VCR. Cases 8, 9, and 12 are separated from this profile by their higher percentage of CCR, diverging from the TD2 group, and a lower percentage of FCD, converging with the TD2 group.

**FIGURE 7:**
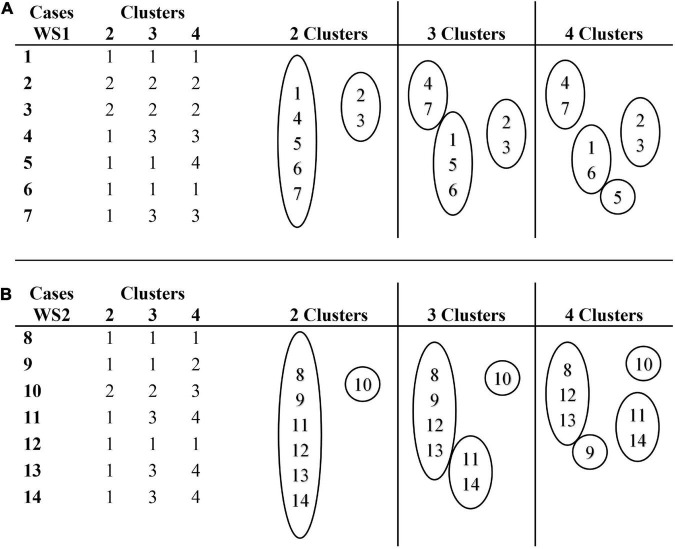
Cluster membership for a range of solutions (2, 3, and 4 clusters) for substitution processes. **(A)** Cluster membership of cases in WS1 group. **(B)** Cluster membership of cases in WS2 group.

In Figure 7, the membership clusters of the RF profiles of SBT subclasses of processes are shown. Figure 7A shows that the profiles of cases 1, 5, and 6 represent the modal profile of the WS1 group. Cases 2 and 3 present an additional profile, which diverges from the TD1 group by its lower percentage of LIQ substitutions and a higher percentage of VLS substitutions; and converges with the TD1 group by its higher percentage of FRC substitutions. Cases 4 and 7 present an additional profile, which diverges from the TD1 group due to its higher percentage of LIQ substitutions.

Figure 7B represents the membership clusters of the WS2 group participants, where it is observed that the profiles of cases 8, 12, and 13 represent the modal profile. Case 10 is an extreme case because of its high percentage of VOW substitutions processes, and case 9 is also an extreme case because it only presents LIQ substitutions. Cases 11 and 14 present an additional profile, which diverges from the TD2 group by the absence of LIQ substitutions and by its higher percentage of FRC substitutions.

**FIGURE 8:**
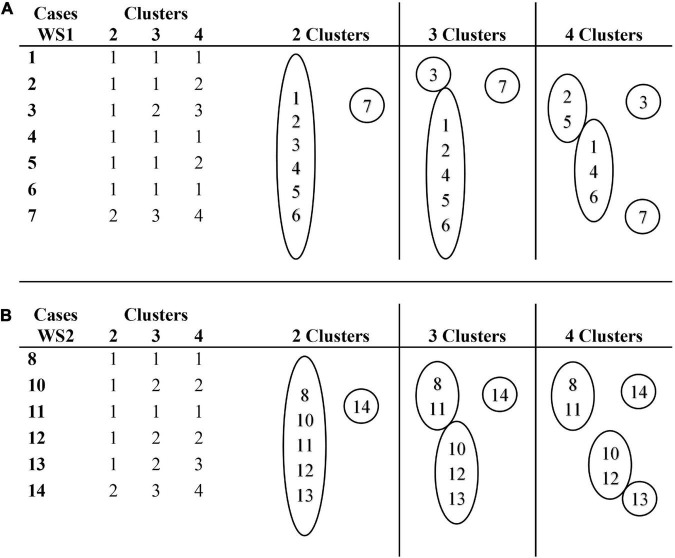
Cluster membership for a range of solutions (2, 3, and 4 clusters) for omission processes. **(A)** Cluster membership of cases in WS1 group. **(B)** Cluster membership of cases in WS2 group.

In Figure 8, the membership clusters of the RF profiles of the OMI subclasses of processes are shown. Figure 8A shows that the profiles of cases 1, 2, 3, 4, 5, and 6 represent the modal profile of the WS1 group. Case 7 is an extreme outlying case because it only presents LIQ omissions processes. Case 3 is also an extreme outlying case, because of its low percentage of LIQ omissions, and its high percentage of VOS, VOW, and FRC omissions.

Figure 8B represents the membership clusters of the WS2 group participants, where it is observed that the profiles of cases 10, 12, and 13 represent the modal profile. Case 14 is an extreme outlying case, because of its high percentage of VOW omissions processes. Cases 8 and 11 present an additional profile, which converges with the TD2 group for its lower percentage of LIQ omissions and its higher percentage of VOS omissions.

## Discussion

The purpose of this study was to explore late phonological development in individuals with WS by comparing the profiles of a group of children (aged 3–8 years) and a group of adolescents and adults (aged 14–25 years). To determine if they followed the stages of typical development and if they presented specific characteristics, they were also compared with the profiles of TD children in two phonological stages: expansion stage (aged 3 years) and resolution stage (aged 5 years). The profiles were based on the classes and subclasses of processes, calculating their PI (frequency of processes/100 words) and their RF (percentage distribution). Additionally, modal profiles and outliers were explored by cluster analysis.

### Stages in late phonological development

The results of the cross-sectional comparison between the group of children and older individuals with WS suggest a late phonological developmental trajectory in which some processes persist into adolescence and adulthood. Children with WS presented a higher frequency of phonological processes in most classes and subclasses than older individuals, which is consistent with previous research (Huffman, 2019). The profiles of both groups were comparable respectively to those of 3- and 5-year-old children in the normative groups, so it could be interpreted that they were in different stages of late phonological development, i.e., the group of children with WS would be in the initial stage of expansion and the group of older individuals would be in the final stage of resolution, according to the chronology established for late phonological development in Spanish (Diez-Itza et al., 2001; Diez-Itza and Martínez, 2004).

The dynamics observed for phonological development also suggest that both groups are at different stages. The frequency of processes in the group of children with WS tended to decrease with chronological age, suggesting that phonological development occurs at a certain rate at this stage, which is not inconsistent with the findings previously reported by Martínez et al. (2014) in two children with WS. This rate of phonological development would compensate for the delay in language onset, which in turn has been related to delayed babbling (Masataka, 2001) and auditory-visual integration difficulties observed in infants and toddlers with WS and other neurodevelopmental syndromes (D’Souza et al., 2015). However, it remains unclear why syndromes follow quite different trajectories of phonological development (Huelmo et al., 2017; Hidalgo and Garayzábal, 2019; Diez-Itza et al., 2021).

In the case of WS, rapid outcomes during the stage of phonological expansion could be favored by an acceleration of lexical development, which initially presents an atypical trajectory where declarative gesture (pointing) is delayed about 6 months in relation to first words. Unlike in typical developing, it is not the onset of first words but the age of acquisition of pointing that best predicts the lexical development of children with WS at 4 years of age, and it also seems to mark the beginning of a necessarily accelerated reconvergence to the trajectory of typical development (Becerra and Mervis, 2019). The recovery of the rate of typical phonological development could be explained in the same way, given the close relationship between lexical and phonological development, and their interrelation with central cognitive processes, such as verbal working memory, reasoning ability, and verbal STM (Mervis et al., 2004; Stoel-Gammon, 2011).

Nevertheless, our results suggest that reconvergence during the expansion stage is not maintained over time, since the phonological profiles in the group of adolescents and adults with WS tended to be progressively divergent compared to those of TD children in the expansion and resolution stages of late phonological development. Moreover, the frequency of phonological processes in these older individuals with WS was not significantly correlated with chronological age and processes persisted in most classes, suggesting that the resolution stage is not completed during adolescence and adulthood in WS. At these ages, asynchronies might be more evident, since phonological production accuracy in older individuals with WS was below that expected for 6-year-old TD children, while their lexical verbal age was close to that expected for 10-year-old TD children, the age at which phonological acquisition can be considered complete.

In any case, the results of the present study indicate that phonology is not fully preserved in WS and should not be considered a relative strength compared to lexical development, as some initial studies had suggested (Udwin and Yule, 1990; Volterra et al., 1996). Similar results have been reported in the case of morphology, which also leads to question its intactness and typicality (Diez-Itza et al., 2017, 2019).

The persistence of phonological processes in adolescents and adults with WS could be related to the atypical phonological processing reported in previous studies (Majerus et al., 2003; Majerus, 2004). In this regard, Huffman (2019) also found that articulatory accuracy was closely associated with phonological processing, intellectual abilities, and lexical abilities. The strength in pseudoword repetition tests, which are at the level expected for chronological age, suggests that STM is not impaired in WS, unlike in Down syndrome (Jarrold and Baddeley, 2001). However, the persistence of processes might be consistent with the hypothesis of a dissociation between short-term and long-term memory in the verbal domain (Vicari et al., 1996b). Previous results, including also phonological awareness tasks, point to more complex cognitive, prosodic, and lexical factors, which determine less finely grained and abnormally structured phonological and lexical representations (Laing et al., 2001; Böhning et al., 2002; Majerus et al., 2003; Stojanovik, 2010). In TD children and adults, links between cognitive and linguistic processing demands and speech motor performance have been identified, whereby the phonological processes observed may also be related to oral-motor difficulties that adolescents and adults with WS still present (Nip et al., 2009; Krishnan et al., 2015).

### Quantitative differences: Frequency of processes

Participants in the WS groups showed a higher frequency of omission than the children in the normative TD groups, including deletion of singletons both in onset syllable positions and in final word coda positions, which may have additional morphophonological developmental implications (Levy and Eilam, 2013). Spanish has a complex morphology where omissions or substitutions of final word sounds may have an impact on inflection in most word categories, especially in verbs. In fact, adolescents with WS presented a higher frequency of morphological omission errors than 5-year-old TD controls matched on verbal age (Diez-Itza et al., 2019).

The children with WS tended to omit all phoneme subclasses more frequently than 3-year-old TD children, although the differences were not statistically significant for voiced stop and liquid consonants, which was unexpected considering that unvoiced stops are less marked and cross-linguistically earlier acquired, i.e., less complex (McLeod and Crowe, 2018). In previous studies, mismatch patterns in tautosyllabic consonant clusters were more common when C1 was voiced (in Spanish, 13 tautosyllabic consonant clusters are possible: /p, t, k, b, d, g, f/ + /liquid/). Voiced stops are more marked and, from a sonority hierarchy approach, closer to C2 liquid consonants, therefore the cluster reduction patterns were considered to follow the principle of retaining the less sonorous consonant (Pérez et al., 2018; Vergara et al., 2021).

The higher frequency of vowel omission observed in the two groups with WS compared to their respective normative TD groups may be considered an atypical feature. In addition, a significantly higher frequency of liquid consonant omissions in the group of adolescents and adults with WS than in the 5-year-old TD normative group may suggest a deviant developmental trajectory. In this group, frequency of omissions of voiceless stop, nasal and fricative consonants may be interpreted as reconverging with the normative group, thus also following a non-linear trajectory of phonological development. The results of this study were partially consistent with those of Diez-Itza et al. (2021) who observed that children and adolescents with Down syndrome presented atypically more omission processes than their 3-year-old TD controls. A substantial portion of the segmental omissions corresponded to codas in medial and final position, which were significantly more frequent in participants with Down syndrome.

The frequency of metathesis was also atypically higher in the group of children with WS compared to the 3-year-old TD children, while in the group of older individuals it no longer differed from the 5-year-old TD children. Early case studies of children with WS have already referred to examples of metathesis as distinctive phonological errors of this syndrome, which was also documented when compared to other syndromes (Volterra et al., 1996; Diez-Itza et al., 1998; Hidalgo, 2019; Hidalgo and Garayzábal, 2019).

It is important to point out the possible effect of the elicitation method, as Diez-Itza et al. (2021) observed that children and adolescents with Down syndrome presented a higher tendency for the omission of segments in spontaneous speech than in articulation tests. Conversely, they found a lower tendency for substitutions in spontaneous speech, consistent with the findings of the present study, where participants with WS did not differ from TD children in consonant substitutions. Nevertheless, they presented a significantly higher frequency of vowel substitutions than TD children, which can be also considered an atypical feature in WS, since single vowels (i.e., simple syllable nuclei) usually appear to be already acquired in Spanish typical late phonological development (Diez-Itza et al., 2001). The striking fact that the study by Hidalgo (2019) did not observe final coda omissions (i.e., final consonant deletions) in any participant with WS also suggests greater facilitation in whole-word structure production when it is elicited through tests of articulation. The tendency for omissions observed in the participants with WS in the present study could therefore be related to the elicitation method, since spontaneous speech involves prosodic, articulatory, and linguistic planning factors quite different from picture naming. Nonetheless, production errors are much less frequent in WS than in Down syndrome, so speech intelligibility is rarely affected in this population, which usually shows a slowed speech rate (Semel and Rosner, 2003; Kumin, 2006; Setter et al., 2007; Crawford et al., 2008; Barnes et al., 2009; Hargrove et al., 2012). In any case, an early and continued speech therapy intervention that addresses specific problems in phonological production of people with WS and an improvement of the home literacy environment, also considering speech rate, should not be omitted (Mervis and Velleman, 2011; Ranzato et al., 2021).

### Qualitative differences: Profiles of percentage distribution of processes

In addition to the quantitative differences observed, the study of relative frequencies further suggests that late phonological development in WS may not follow an entirely linear trajectory. Intersections between the profiles of relative frequency between the groups of children and older individuals with WS might suggest that the trajectories from the first stage to the final stage of late phonological development is toward reduction in the proportion of omissions and increase in the proportion of assimilation and addition processes. Such hypothesized trajectories are in line with the observed differences between TD normative groups and, therefore, the profiles of adolescents and adults with WS and 5-year-old TD children appeared to be quite close, suggesting that the trajectory observed in WS would correspond to the typical evolution from the expansion stage to the resolution stage in late phonological development. These results are consistent in part with those of Martínez and Diez-Itza (2012), who observed that assimilations tended to persist as errors of processing in the last stage of typical development.

However, the profiles of relative frequency presented an atypically higher percentage of omissions in the group of children with WS than in the normative group of 3-year-old TD children. Accordingly, the percentage of substitutions in the WS1 group was low, so that the profiles of both groups intersected at those points, suggesting that the children with WS may still be in an earlier stage, since in typical late phonological development an emergent process is observed in which substitutions tend to increase and omissions to decrease during the expansion stage (Vergara et al., 2021).

Differences became more apparent in the profiles of the subclasses of processes. Syllable structure subclasses showed intersecting profiles in the WS groups, with a relatively lower percentage of final consonant deletion and a relatively higher percentage of vowel cluster reduction (diphthongs) in the group of adolescents and adults with WS, which is in line with the profiles of the TD normative groups in the respective stages of expansion and resolution.

However, striking asymmetries were also found in the profiles of the WS groups when compared with the TD groups: the WS children presented a much lower percentage of consonant cluster reduction than the 3-year-old TD children, which contrasts with the high percentage of final consonant deletion. It is important to note that the present study as the previous one by Martínez (2010) included both tautosyllabic and heterosyllabic consonant clusters, which were also fully described in Diez-Itza and Martínez (2004). In contrast, a more recent study including non-linear analyses, where a brief description of the Spanish phonological system can be found, focused only in tautosyllabic clusters (Vergara et al., 2021). The observed profile of early acquisition of consonant clusters indicates an asynchronous development since the accurate production of consonant clusters is typically protracted in late phonological development and it is commonly impaired in speech disorders (McLeod et al., 2001; Pérez et al., 2018; Vergara et al., 2021). The profile of adolescents and adults with WS reconverges in this respect with that of the group of 5-year-old TD children, suggesting an atypical trajectory (Becerra and Mervis, 2019). However, the persistent deletion of final consonants remains a divergent feature in the profile of older individuals with WS, and this should be investigated in relation to the atypical morphophonological difficulties noted in some studies (Levy and Eilam, 2013; Diez-Itza et al., 2017).

There were also marked differences in the profiles when the relative frequencies of substitutions and omissions were analyzed. In the group of adolescents and adults with WS, a lower percentage was observed in the substitutions of voiceless stops, with a higher percentage of processes in voiced phonemes, in concordance with a typical trajectory also observed in the profiles of the TD controls (Vergara et al., 2021). The profiles of children with WS showed a higher percentage of vowel substitutions than those of 3-year-old TD children, which might be considered an atypical feature, as studies suggest that single vowels are acquired in the early stages of phonological development (Smith, 1973; Bosch, 2004). A relatively high percentage of vowel substitution processes is maintained in the WS2 group, although it also corresponds to a relative increase of vowel substitutions in the normative TD2 group. This observation is consistent with that of Donegan (2013), who suggests that there is greater vowel variability in children than is usually considered and this is explained by both phonetic and prosodic factors. It seems that vowels play a different role than consonants in language acquisition and they are related to prosody and the organization of syntactic constituents (Hochmann et al., 2011), so vowel substitution processes may be associated with the prosodic difficulties observed in WS (Stojanovik, 2010; Martínez-Castilla et al., 2012).

Regarding the subclasses of omission processes, the profiles of older individuals with WS showed significant reductions in the percentages of omission of voiceless stop, nasal, and fricative consonants, suggesting non-linear trajectories across stages of phonological development. In addition, the profiles of children with WS showed a lower percentage of fricative consonant omissions and higher percentages of omission of single vowels, voiceless stops, and nasals than those observed in the normative 3-year-old TD group, which again points to atypical features in WS late phonological development. The older individuals with WS presented a profile of relative frequency of omissions that also diverges from that of TD 5-year-old children, where higher percentages of liquid consonant and single vowel omissions were observed.

The results of the present study therefore reveal that, beyond the observed parallels, which suggested different stages and non-linear trajectories in late phonological development in both WS and TD, partially deviant profiles also appear when comparing the relative frequencies of the processes of the WS groups and their respective normative TD groups. These qualitative differences could be interpreted as atypical patterns in the profiles of individuals with WS with respect to what would be expected based on the stages of phonological development. Thus, phonological development across late stages might not be explained merely as a delay, i.e., only in terms of quantitative differences in frequency of processes based on chronological age. Atypical trajectories of development in individuals with WS and cross-syndrome differences have also been described at other levels of language such as morphology, prosody, lexical abilities, and pragmatics (Thomas and Karmiloff-Smith, 2003; Levy and Eilam, 2013; Diez-Itza et al., 2019, 2022).

Furthermore, adolescents and adults with WS, while they are in some respects at the same stage of resolution as 5-year-old TD children, exhibit an asynchronous and atypical persistence of certain processes suggesting that they have completed late phonological development without full mastery of phonology. This may be due to atypical phonological processing, inaccurate representations in long-term memory, or factors related to oral-motor development that require further investigation (Laing et al., 2001; Böhning et al., 2002; Majerus et al., 2003; Majerus, 2004, Nip et al., 2009; Krishnan et al., 2015).

### Individual differences and modal profiles: Cluster analyses

The individual profiles based on relative frequencies of processes were compared by cluster analysis and it was observed that most individuals with WS presented modal profiles, i.e., adjusted to the mean of their group, for the different classes of processes. However, important individual differences also emerged, as previous studies had observed in conversations of people with WS (Stojanovik, 2006). These differences were in the direction of greater divergence from the profiles of the normative TD groups, expanding the atypical features of late phonological development in WS. Moreover, modal profiles were not always represented in the different classes of processes by the same participants, indicating a great complexity where individual differences interact with developmental trajectories.

In the more detailed analysis of the differences and similarities in the individual profiles of the subclasses of processes, it was observed that most of the children with WS presented a modal profile in syllable structure and omission processes. However, the case of the oldest participant in this group was an outlier in both classes of processes, diverging from the profiles of the normative TD group and the older participants with WS. She also diverged from the group in the profile of substitution processes, being the only case outside the modal profile in all classes and subclasses of processes. This may be interpreted as suggesting that because of her older chronological and verbal age she no longer represents the first stage of late phonological development in WS, but perhaps the intermediate stage of stabilization that was not captured in the present study.

The group of adolescents and adults with WS showed greater heterogeneity so that, in the profiles of all the subclasses of processes, the modal group did not include a majority of cases. This could be related to the fact that they are at a different stage of development and have a wider range of chronological age and verbal age. Among the extreme cases, the most outstanding were: the one with the highest verbal age who showed very atypical profiles of syllable structure and substitution processes; the one with the highest chronological age and the lowest verbal age, with a high proportion of vowel omission processes, typical of earlier stages; and the only case that was not included in any of the modal profiles and that also only presented substitution of liquid consonants. These results suggest that verbal age is a factor that may determine not only quantitative differences in phonological production but also greater complexity and qualitative differences in the classes and subclasses of processes. However, non-verbal abilities, gender, or schooling could also account for individual differences.

### Limitations of the study

It is necessary to recognize several limitations in the present study. The sample size was small due to the difficulty of recruiting participants with this relatively rare syndrome and of applying a naturalistic methodology, more complex than the use of articulation tests, although it might be considered sufficient for a first exploratory study, taking into account that the word samples analyzed were large (more than 40,000 word tokens, and almost 10,000 word types). As shown by the analyses of individual differences, the chronological and verbal age ranges are too wide and not having separated groups for adolescents and adults is also a limitation. Future studies should better adjust the age of the groups and exclude atypical cases. The computation of the frequency of processes on the total number of tokens, instead of on the total number of word types, although providing control over the size of the individual samples analyzed, may in some cases overestimate the phonological index, computing the same error several times, or underestimate it, in those cases with more lexical diversity. Although the procedure followed in transcription and coding assured a high level of interrater reliability, the study lacked a numerical index to properly account for this potential source of error. Since the elicitation method could influence the results, it should be recommended to combine spontaneous speech assessment with articulation tests in future studies. Non-verbal abilities (e.g., short and long-term memory) and other factors such as word frequency and word length also may have had effects on phonological production that were not controlled for in this study, although it should be noted that the use of the words in spontaneous speech guarantees that they are part of the vocabulary of the participants.

## Conclusion

The present exploratory study of late phonological development in WS suggested that children between 3 and 8 years of age and adolescents and adults between 14 and 25 years of age are at different stages of late phonological development. The frequency of phonological processes of the group of children with WS was comparable to that of 3-year-old TD children, implying that both would be at the same first stage of late phonological development (namely, the expansion stage). Older individuals with WS presented a much lower frequency of processes, similar to that of 5-year-olds in the last stage of phonological development (namely, the resolution stage). However, phonology no longer seems to be developing in the adolescents and adults with WS, whose phonological processes would therefore be persistent and independent of chronological age. Moreover, their marked age asynchrony of more than fourteen years with the TD normative group does not make it suitable to describe these persistent phonological difficulties in terms of delayed or protracted phonological development, nor the fact that they presented a frequency of phonological processes above that expected for their verbal lexical age. In contrast, children with WS showed a certain rate of phonological development that tends to bring them closer to the level expected for their verbal age, although with an asynchrony of almost 3 years below their chronological age.

These asynchronies are associated with atypical features in the phonology of individuals with WS that were revealed in both quantitative (frequency) and qualitative (proportion) assessments of phonological processes. Although the profiles were partially coincident with those of TD children, they also presented specific features, which were more evident when the subclasses of processes were analyzed in detail. The analysis of the underlying processes, especially in substitutions and omissions, revealed specific and complex phonological profiles in both groups of participants with WS. Individual differences tended to increase the divergence from the typical developmental profiles, being more salient in the group of adolescents and adults with WS, although participants who suited the average profile of the group predominated. The greater tendency to omissions in all syllable positions, including final codas, can be considered atypical and characteristic of WS at all ages, and may also be related to morphological processes. However, it is possible that this finding was in part influenced by the elicitation method based on spontaneous speech, as has been observed in Down syndrome.

The results of this study, although requiring further research, provide some new insight into atypical and dynamic phonological developmental trajectories in WS. Chronological and verbal age account for individual differences in phonological production, although other variables including short and long-term memory should be analyzed in future studies. The findings may also have clinical implications for speech intervention in this population requiring continued specific assessment and treatments adapted to the emerging characteristics of their phonological profiles throughout childhood, adolescence, and into adulthood.

## Data availability statement

The raw data supporting the conclusions of this article will be made available by the authors, without undue reservation.

## Ethics statement

The studies involving human participants were reviewed and approved by the Ethical Committee in Research of the University of Oviedo. Written informed consent to participate in this study was provided by the participants or their legal guardian/next of kin.

## Author contributions

ED-I had a primary role in the conception and design of the study, development of the coding scheme, data analysis and discussion, and drafting of the manuscript. VP and VM helped with the design and conducted the research, carried out the transcription, coding, data analyses, and helped draft the manuscript. All authors contributed to the article and approved the submitted version.

## References

[B1] Abbeduto L. Benson G. Short K. Dolish J. (1995). Effects of sampling context on the expressive language of children and adolescents with mental retardation. *Ment. Retard.* 33 279–288. 7476250

[B2] Aguilar E. Serra M. (2003). *A-RE-HA: Análisis del retraso del habla: Protocolos para el análisis de la fonética y la fonología infantil.* Barcelona: Universitat de Barcelona.

[B3] Barnes E. Roberts J. Long S. H. Martin G. E. Berni M. C. Mandulak K. C. (2009). Phonological accuracy and intelligibility in connected speech of boys with Fragile X syndrome or Down syndrome. *J. Speech Lang. Hear. Res.* 52 1048–1061. 10.1044/1092-4388(2009/08-0001) 19641081 PMC2719827

[B4] Becerra A. M. Mervis C. B. (2019). Age at onset of declarative gestures and 24-month expressive vocabulary predict later language and intellectual abilities in young children with Williams syndrome. *Front. Psychol.* 10:2648. 10.3389/fpsyg.2019.02648 31849765 PMC6901496

[B5] Bellugi U. Lichtenberger L. Jones W. Lai Z. St. George M. (2000). I. The neurocognitive profile of Williams syndrome: A complex pattern of strengths and weaknesses. *J. Cogn. Neurosci.* 12 (Suppl. 1), 7–29. 10.1162/089892900561959 10953231

[B6] Bellugi U. Marks S. Bihrle A. Sabo H. (1988). “Dissociation between language and cognitive functions in Williams syndrome,” in *Language development in exceptional circumstances*, eds Bishop D. Mogford K. (London: Churchill Livingstone), 177–189.

[B7] Bellugi U. Wang P. P. Jernigan T. L. (1994). “Williams syndrome: An unusual neuropsychological profile,” in *Atypical cognitive deficits in developmental disorders: Implications for brain function*, eds Broman S. Grafman K. (Hillsdale, NJ: Lawrence Erlbaum Associates, Inc), 23–56.

[B8] Benítez-Burraco A. Garayzábal E. Cuetos F. (2017). Morphology in Spanish-speaking children with Williams syndrome. *Lang. Cogn.* 9 728–740. 10.1017/langcog.2017.6 26967348

[B9] Bernhardt B. M. Stemberger J. P. (2017). “Investigating typical and protracted phonological development across languages,” in *Crosslinguistic encounters in language acquisition*, eds Babatsouli E. Ingram D. Müller N. (Bristol: Multilingual Matters), 71–108. 10.21832/9781783099092-008

[B10] Böhning M. Campbell R. Karmiloff-Smith A. (2002). Audiovisual speech perception in Williams syndrome. *Neuropsychologia* 40 1396–1406. 10.1016/S0028-3932(01)00208-1 11931944

[B11] Boloh Y. Ibernon L. (2010). Gender attribution and gender agreement in 4- to 10-year-old French children. *Cogn. Dev.* 25 1–25. 10.1016/j.cogdev.2009.09.011

[B12] Bosch L. (2004). *Evaluación fonológica del habla infantil.* MASSON.

[B13] Brock J. (2007). Language abilities in Williams syndrome: A critical review. *Dev. Psychopathol.* 19 97–127. 10.1017/S095457940707006X 17241486

[B14] Camp J. S. Karmiloff-Smith A. Thomas M. S. C. Farran E. K. (2016). Cross-syndrome comparison of real-world executive functioning and problem solving using a new problem-solving questionnaire. *Res. Dev. Disabil.* 59 80–92. 10.1016/j.ridd.2016.07.006 27521717

[B15] Capirci O. Sabbadini L. Volterra V. (1996). Language development in Williams syndrome: A case study. *Cogn. Neuropsychol.* 13 1017–1039. 10.1207/S15326942DN1802_4 11280965

[B16] Clahsen H. Ring M. Temple C. (2004). “Lexical and morphological skills in English-speaking children with Williams syndrome,” in *Language acquisition and language disorders*, Vol. 36 eds Bartke S. Siegmüller J. (Amsterdam: John Benjamins Publishing Company), 221–244. 10.1075/lald.36.14cla

[B17] Cohen J. (1988). *Statistical Power analysis for the behavioral sciences.* Hillsdale, NJ: Lawrence Erlbaum Associates.

[B18] Crawford N. A. Edelson L. R. Skwerer D. P. Tager-Flusberg H. (2008). Expressive language style among adolescents and adults with Williams syndrome. *Appl. Psycholinguist.* 29 585–602. 10.1017/S0142716408080259

[B19] Davis B. L. Bedore L. M. (2013). *An emergence approach to speech acquisition.* Hove: Psychology Press. 10.4324/9780203375303

[B20] Diez-Itza E. (1992). *Adquisición del lenguaje.* Oviedo: Pentalfa.

[B21] Diez-Itza E. Antón A. Fernández-Toral J. García M. L. (1998). “Language development in Spanish children with Williams syndrome,” in *Perspectives on language acquisition*, eds Aksu-Koç A. Erguvanli-Taylan E. Sumru A. Küntay A. (Beşiktaş: Bogazici University), 309–324.

[B22] Diez-Itza E. Martínez V. (2004). Las etapas tardías de la adquisición fonológica: Procesos de reducción de grupos consonánticos. *Anuario Psicol.* 35 177–202.

[B23] Diez-Itza E. Martínez V. Cantora R. Justicia F. Bosch L. (2001). “Late phonological processes in the acquisition of Spanish,” in *Research on child language acquisition*, eds Almgren M. Barreña A. Ezeizabarrena M. J. Idiazábal I. MacWhinney B. (Somerville, MA: Cascadilla Press), 790–799.

[B24] Diez-Itza E. Martínez V. Fernández-Urquiza M. Antón A. (2017). “Morphological profile of Williams syndrome: Typical or atypical?,” in *Language development and disorders in spanish-speaking children*, Vol. 14 eds Auza Benavides A. Schwartz R. G. (Cham: Springer International Publishing), 311–327. 10.1007/978-3-319-53646-0_15

[B25] Diez-Itza E. Martínez V. Miranda M. Antón A. Ojea A. I. Fernández-Urquiza M. (2014). “The Syndroling Project: A comparative linguistic analysis of typical development profiles and neurodevelopmental genetic syndromes (Down, Williams and fragile X syndromes),” in *Proceedings of the IASCL-XII international congress for the study of child language*, Amsterdam.

[B26] Diez-Itza E. Martínez V. Pérez V. Fernández-Urquiza M. (2018). Explicit oral narrative intervention for students with Williams syndrome. *Front. Psychol.* 8:2337. 10.3389/fpsyg.2017.02337 29379455 PMC5775294

[B27] Diez-Itza E. Miranda M. Pérez V. Martínez V. (2019). “Profiles of grammatical morphology in Spanish-speaking adolescents with Williams syndrome and Down syndrome,” in *Atypical language development in romance languages*, eds Aguilar-Mediavilla E. Buil-Legaz L. López-Penadés R. Sanchez-Azanza V. A. Adrover-Roig D. (Amsterdam: John Benjamins Publishing Company), 219–234. 10.1075/z.223.13die

[B28] Diez-Itza E. Snow C. MacWhinney B. (1999). La metodología RETAMHE y el proyecto CHILDES: Breviario para la codificación y análisis de lenguaje infantil. *Psicotema* 11 517–530.

[B29] Diez-Itza E. Vergara P. Barros M. Miranda M. Martínez V. (2021). Assessing phonological profiles in children and adolescents with Down syndrome: The effect of elicitation methods. *Front. Psychol.* 12:662257. 10.3389/fpsyg.2021.662257 34054666 PMC8149804

[B30] Diez-Itza E. Viejo A. Fernández-Urquiza M. (2022). Pragmatic profiles of adults with Fragile X syndrome and Williams syndrome. *Brain Sci.* 12:385. 10.3390/brainsci12030385 35326341 PMC8946534

[B31] Donegan P. (2013). “Normal vowel development,” in *Handbook of vowels and vowel disorders*, eds Ball M. J. Gibbon F. E. (London: Psychology Press), 24–60.

[B32] D’Souza D. Cole V. Farran E. K. Brown J. H. Humphreys K. Howard J. (2015). Face processing in Williams syndrome is already atypical in infancy. *Front. Psychol.* 6:760. 10.3389/fpsyg.2015.00760 26124729 PMC4466450

[B33] D’Souza D. D’Souza H. Jones E. J. H. Karmiloff-Smith A. (2020). Attentional abilities constrain language development: A cross-syndrome infant/toddler study. *Dev. Sci.* 23:e12961. 10.1111/desc.12961 32154971

[B34] Dunn L. M. Dunn L. M. Arribas D. (2010). *PPVT-III PEABODY*. *Test de vocabulario en imágenes.* Madrid: TEA ediciones.

[B35] Garayzábal E. Osório A. Lens M. Sampaio A. (2014). Concrete and relational vocabulary: Comparison between Williams and Smith–Magenis syndromes. *Res. Dev. Disabil.* 35 3365–3371. 10.1016/j.ridd.2014.07.055 25194511

[B36] Grant J. Karmiloff-Smith A. Gathercole S. A. Paterson S. Howlin P. Davies M. (1997). Phonological short-term memory and its relationship to language in Williams syndrome. *Cogn. Neuropsychiatry* 2 81–99. 10.1080/135468097396342 25420198

[B37] Grunwell P. (1981). The development of phonology: A descriptive profile. *First Language* 3 161–191.

[B38] Hargrove P. M. Pittelko S. Fillingane E. Rustman E. Lund B. (2012). Perceptual speech and paralinguistic skills of adolescents with Williams syndrome. *Commun. Disord. Q.* 34 152–161. 10.1177/1525740112436372

[B39] Heiz J. Barisnikov K. (2016). Visual-motor integration, visual perception and motor coordination in a population with Williams syndrome and in typically developing children: Visual-motor abilities in a population with Williams syndrome. *J. Intellect. Disabil. Res.* 60 945–955. 10.1111/jir.12328 27545961

[B40] Hidalgo I. (2019). *El nivel fónico de la población con síndrome de Smith Magenis: Particularidades fonatorias y fonético-fonológicas. Comparativa con síndrome de Williams, síndrome de Down y desarrollo típico.* Ph.D. thesis. Madrid: Universidad Autónoma de Madrid.

[B41] Hidalgo I. Garayzábal E. (2019). Diferencias fonológicas entre síndromes del neurodesarrollo: Evidencias a partir de los procesos de simplificación fonológica más frecuentes. *Rev. Invest. Logopedia* 9 81–106. 10.5209/rlog.62942

[B42] Hochmann J.-R. Benavides-Varela S. Nespor M. Mehler J. (2011). Consonantsand vowels: Different roles in early language acquisition. *Dev. Sci*. 14, 1445–1458. 10.1111/j.1467-7687.2011.01089.x 22010902

[B43] Huelmo J. Martínez V. Diez-Itza E. (2017). Evaluación de perfiles fonológicos en el síndrome X-Frágil mediante índices de error. *Rev. INFAD Psicol.* 4 67–76. 10.17060/ijodaep.2017.n1.v4.1028

[B44] Huffman M. J. (2019). *Speech articulation in children with Williams syndrome or 7q11.23 duplication syndrome.* Ph.D. thesis. Louisville, KY: University of Louisville.

[B45] Ingram D. (1976). *Phonological disability in children.* London: Edward Arnold.

[B46] Jarrold C. Baddeley A. D. (2001). Short-term memory in Down syndrome: Applying the working memory model. *Downs Syndr. Res. Pract.* 7 17–23. 10.3104/reviews.110 11706808

[B47] Jarrold C. Baddeley A. D. Hewes A. K. Phillips C. (2001). A Longitudinal assessment of diverging verbal and non-verbal abilities in the Williams syndrome phenotype. *Cortex* 37 423–431. 10.1016/S0010-9452(08)70583-5 11485066

[B48] Järvinen A. Korenberg J. R. Bellugi U. (2013). The social phenotype of Williams syndrome. *Curr. Opin. Neurobiol.* 23 414–422. 10.1016/j.conb.2012.12.006 23332975 PMC4326252

[B49] Karmiloff-Smith A. (1998). Development itself is the key to understanding developmental disorders. *Trends Cogn. Sci.* 2 389–398. 10.1016/S1364-6613(98)01230-3 21227254

[B50] Karmiloff-Smith A. Grant J. Berthoud I. Davies M. Howlin P. Udwin O. (1997). Language and Williams syndrome: How intact is “intact”? *Child Dev.* 68 246–262. 9180000

[B51] Kirchner R. M. Martens M. A. Andridge R. R. (2016). Adaptive behavior and development of infants and toddlers with Williams syndrome. *Front. Psychol.* 7:598. 10.3389/fpsyg.2016.00598 27199832 PMC4848290

[B52] Kozel B. A. Barak B. Kim C. A. Mervis C. B. Osborne L. R. Porter M. (2021). Williams syndrome. *Nat. Rev. Dis. Primers* 7:42. 10.1038/s41572-021-00276-z 34140529 PMC9437774

[B53] Krishnan S. Bergström L. Alcock K. J. Dick F. Karmiloff-Smith A. (2015). Williams syndrome: A surprising deficit in oromotor praxis in a population with proficient language production. *Neuropsychologia* 67 82–90. 10.1016/j.neuropsychologia.2014.11.032 25433223 PMC4410792

[B54] Kumin L. (2006). Speech intelligibility and childhood verbal apraxia in children with Down syndrome. *Down Syndr. Res. Pract.* 10 10–22. 10.3104/reports.301 16869369

[B55] Laing E. Hulme C. Grant J. Karmiloff-Smith A. (2001). Learning to read in Williams syndrome: Looking beneath the surface of atypical reading development. *J. Child Psychol. Psychiatry* 42 729–739. 10.1111/1469-7610.00769 11583245

[B56] Levy Y. Eilam A. (2013). Pathways to language: A naturalistic study of children with Williams syndrome and children with Down syndrome. *J. Child Lang.* 40 106–138. 10.1017/S0305000912000475 23217293

[B57] MacWhinney B. (2000). *The CHILDES project: Tools for analyzing talk.Transcription format and programs*, Vol. I. Hillsdale, NJ: Lawrence Erlbaum.

[B58] Majerus S. (2004). “Phonological processing in Williams syndrome,” in *Language Acquisition and Language Disorders*, Vol. 36 eds Bartke S. Siegmüller J. (Amsterdam: John Benjamins Publishing Company), 125–142. 10.1075/lald.36.10maj

[B59] Majerus S. Barisnikov K. Vuillemin I. Poncelet M. Linden M. (2003). An investigation of verbal short-term memory and phonological processing in four children with Williams syndrome. *Neurocase* 9 390–401. 10.1076/neur.9.5.390.16558 14972754

[B60] Martínez V. (2010). *Etapas tardías del desarrollo fonológico infantil: Procesos y límites del trastorno.* Ph.D. thesis. Oviedo: Universidad de Oviedo.

[B61] Martínez V. Antón A. Miranda M. Pérez V. Fernández-Toral J. Diez-Itza E. (2014). “Accelerated phonological development in Williams syndrome: A two case corpus-based study of late phonological processes,” in *Proceedings of the IASCL-XII international congress for the study of child language*, Amsterdam.

[B62] Martínez V. Diez-Itza E. (2012). Procesos de asimilación en las etapas tardías del desarrollo. *Psicothema* 24 193–198. 22420344

[B63] Martínez-Castilla P. Stojanovik V. Setter J. Sotillo M. (2012). Prosodic abilities in Spanish and English children with Williams syndrome: A cross-linguistic study. *Appl. Psycholinguist.* 33 1–22. 10.1017/S0142716411000385

[B64] Masataka N. (2001). Why early linguistic milestones are delayed in children with Williams syndrome: Late onset of hand banging as a possible rate–limiting constraint on the emergence of canonical babbling. *Dev. Sci.* 4 158–164. 10.1111/1467-7687.00161

[B65] Mayall L. A. D’Souza H. Hill E. L. Karmiloff-Smith A. Tolmie A. Farran E. K. (2021). Motor abilities and the motor profile in individuals with Williams syndrome. *Adv. Neurodev. Disord.* 5 46–60. 10.1007/s41252-020-00173-8

[B66] McLeod S. Crowe K. (2018). Children’s consonant acquisition in 27 languages: A cross-linguistic review. *Am. J. Speech Lang. Pathol.* 27 1546–1571. 10.1044/2018_AJSLP-17-0100 30177993

[B67] McLeod S. Van Doorn J. Reed V. (2001). Consonant cluster development in two-year-olds: General trends and individual difference. *J. Speech Lang. Hear. Res.* 44 1144–1171. 10.1044/1092-4388(2001/090) 11708533

[B68] Mervis C. B. Becerra A. M. (2007). Language and communicative development in Williams syndrome. *Ment. Retard. Dev. Disabil. Res. Rev.* 13 3–15. 10.1002/mrdd.20140 17326109

[B69] Mervis C. B. John A. E. (2008). Vocabulary abilities of children with Williams syndrome: Strengths, weaknesses, and relation to visuospatial construction ability. *J. Speech Lang. Hear. Res.* 51 967–982. 10.1044/1092-4388(2008/071) 18658065 PMC2562689

[B70] Mervis C. B. Klein-Tasman B. P. Huffman M. J. Velleman S. L. Pitts C. H. Henderson D. R. (2015). Children with 7q11.23 duplication syndrome: Psychological characteristics. *Am. J. Med. Genet. A* 167 1436–1450. 10.1002/ajmg.a.37071 25900101 PMC4545595

[B71] Mervis C. B. Robinson B. F. Rowe M. L. Becerra A. M. Klein-Tasman B. P. (2004). “Relations between language and cognition in Williams syndrome,” in *Language acquisition and language disorders*, Vol. 36 eds Bartke S. Siegmüller J. (Amsterdam: John Benjamins Publishing Company), 63–92. 10.1075/lald.36.08mer

[B72] Mervis C. B. Velleman S. L. (2011). Children with Williams syndrome: Language, cognitive, and behavioral characteristics and their implications for intervention. *Perspect. Lang. Learn. Educ.* 18 98–107. 10.1044/lle18.3.98 22754603 PMC3383614

[B73] Miezah D. Porter M. Batchelor J. Boulton K. Campos G. (2020). Cognitive abilities in Williams syndrome. *Res. Dev. Disabil.* 104:103701. 10.1016/j.ridd.2020.103701 32554266

[B74] Moraleda E. López P. (2020). Analysis of receptive vocabulary development in Williams syndrome. *Open J. Mod. Linguist.* 10 804–812. 10.4236/ojml.2020.106050

[B75] Nip I. S. B. Green J. R. Marx D. B. (2009). Early speech motor development: Cognitive and linguistic considerations. *J. Commun. Disord.* 42 286–298. 10.1016/j.jcomdis.2009.03.008 19439318 PMC2892123

[B76] Pérez D. Vivar P. Bernhardt B. M. Mendoza E. Ávila C. Carballo G. (2018). Word-initial Rhotic clusters in Spanish-speaking preschoolers in Chile and Granada, Spain. *Clin. Linguist. Phon.* 32 481–505. 10.1080/02699206.2017.1359852 28956653

[B77] Pérez Jurado L. A. (2003). Williams-Beuren syndrome: A model of recurrent genomic mutation. *Horm. Res. Paediatr.* 59 106–113. 10.1159/000067836 12638521

[B78] Pérez-García D. Brun-Gasca C. Pérez-Jurado L. A. Mervis C. B. (2017). Behavioral profiles of children with Williams syndrome from Spain and the United States: Cross-Cultural similarities and differences. *Am. J. Intellect. Dev. Disabil.* 122 156–172. 10.1352/1944-7558-122.2.156 28257245 PMC5339738

[B79] Purser H. R. M. Thomas M. S. C. Snoxall S. Mareschal D. Karmiloff-Smith A. (2010). Definitions versus categorization: Assessing the development of lexico-semantic knowledge in Williams syndrome. *Int. J. Lang. Commun. Disord.* 46 361–373. 10.3109/13682822.2010.497531 21575076

[B80] Ranzato E. Tolmie A. Van Herwegen J. (2021). The home learning environment of primary school children with Down Syndrome and those with Williams Syndrome. *Brain Sci.* 11:733. 10.3390/brainsci11060733 34073060 PMC8229284

[B81] Reilly J. Losh M. Bellugi U. Wulfeck B. (2004). Frog, where are you?” Narratives in children with specific language impairment, early focal brain injury, and Williams syndrome. *Brain Lang.* 88 229–247. 10.1016/S0093-934X(03)00101-9 14965544

[B82] Rose Y. Inkelas S. (2011). “The interpretation of phonological patterns in first language acquisition: The interpretation of phonological patterns in first language acquisition,” in *The Blackwell companion to phonology*, eds van Oostendorp M. Ewen C. J. Hume E. Rice K. (Malden, MA: Wiley-Blackwell), 1–25. 10.1002/9781444335262.wbctp0101

[B83] Semel E. Rosner S. R. (2003). *Understanding Williams syndrome.* Mahwah, NJ: Lawrence Erlbaum Associates. 10.4324/9781410607416

[B84] Setter J. Stojanovik V. Van Ewijk L. Moreland M. (2007). Affective prosody in children with Williams syndrome. *Clin. Linguist. Phon.* 21 659–672. 10.1080/02699200701539056 17701754

[B85] Smith N. V. (1973). *The acquisition of phonology. A case study*. Cambridge: Cambridge University Press.

[B86] Stoel-Gammon C. (2011). Relationships between lexical and phonological development in young children. *J. Child Lang.* 38 1–34. 10.1017/S0305000910000425 20950495

[B87] Stojanovik V. (2006). Social interaction deficits and conversational inadequacy in Williams syndrome. *J. Neurolinguist.* 19 157–173. 10.1016/j.jneuroling.2005.11.005

[B88] Stojanovik V. (2010). Understanding and production of prosody in children with Williams syndrome: A developmental trajectory approach. *J. Neurolinguist.* 23 112–126. 10.1016/J.JNEUROLING.2009.11.001

[B89] Stojanovik V. Perkins M. Howard S. (2001). Language and conversational abilities in Williams syndrome: How good is good? *Int. J. Lang. Commun. Disord.* 36 234–239. 10.3109/13682820109177890 11340788

[B90] Thomas M. S. C. Grant J. Barham Z. Gsödl M. Laing E. Lakusta L. (2001). Past tense formation in Williams syndrome. *Lang. Cogn. Process.* 16 143–176. 10.1080/01690960042000021

[B91] Thomas M. S. C. Karmiloff-Smith A. (2003). Modeling language acquisition in atypical phenotypes. *Psychol. Rev.* 110 647–682. 10.1037/0033-295X.110.4.647 14599237

[B92] Udwin O. Yule W. (1990). Augmentative communication systems taught to cerebral palsied children-a longitudinal study. I. The acquisition of signs and symbols, and syntactic aspects of their use over time. *Int. J. Lang. Commun. Disord.* 25 295–309. 10.3109/13682829009011979 2095837

[B93] Velleman S. L. Currier A. Caron T. Curley A. Mervis C. B. (2006). *Phonological development in Williams syndrome.* (Dubrovnik: International Clinical Phonetics and Linguistics Association), 108–116.

[B94] Vergara P. Bernhardt B. M. Pérez D. Diez-Itza E. (2021). Consonant cluster acquisition in Chilean children with typical and protracted phonological development. *Clin. Linguist. Phon.* 35 964–982. 10.1080/02699206.2020.1851306 33251868

[B95] Vicari S. Brizzolara D. Carlesimo G. A. Pezzini G. Volterra V. (1996a). Memory abilities in children with Williams syndrome. *Cortex* 32 503–514. 10.1016/S0010-9452(96)80007-4 8886525

[B96] Vicari S. Carlesino G. Brizzolara D. Pezzini G. (1996b). Short-term memory in children with Williams syndrome: A reduced contribution of lexical-semantic knowledge to word span. *Neuropsychologia* 34 919–925. 10.1016/0028-3932(96)00007-3 8822738

[B97] Volterra V. Capirci O. Pezzini G. Sabbadini L. Vicari S. (1996). Linguistic abilities in Italian children with Williams syndrome. *Cortex* 32 663–677. 10.1016/S0010-9452(96)80037-2 8954245

